# Investigating hypoxia-inducible factor signaling in cancer: Mechanisms, clinical implications, targeted therapeutic strategies, and resistance

**DOI:** 10.1016/j.cpt.2025.07.003

**Published:** 2025-07-12

**Authors:** Abdul Halim Shaikat, S.M. Asadul Karim Azad, Md Azizur Rahman Tamim, Mohammed Sailim Ullah, Mohammad Nurul Amin, Mofazzal K. Sabbir, Md Towhidul Islam Tarun, Md Saqline Mostaq, Shohana Sabrin, Md Zihad Mahmud, Md Ashiq Mahmud

**Affiliations:** a Department of Pharmacy, Faculty of Science and Engineering, International Islamic University Chittagong Chattogram, 4318, Bangladesh; b Pratyasha Health Biomedical Research Center, Dhaka 1230, Bangladesh; c Department of Pharmacy, Faculty of Life and Earth Science, Jagannath University, Dhaka 1100, Bangladesh; d Department of Pharmacy, State University of Bangladesh, Dhaka 1461, Bangladesh; e Department of Biology, Indiana State University, Terre Haute, IN 47809, USA; f M Abdur Rahim Medical College, Dinajpur 5200, Bangladesh; g Pioneer Dental College and Hospital, Dhaka 1229, Bangladesh; h School of Basic Pharmaceutical and Toxicological Sciences, College of Pharmacy, University of Louisiana at Monroe, Monroe, LA 71209-0497, USA

**Keywords:** Hypoxia, Drug resistance, Molecular mechanisms, HIF-Related factor, Angiogenesis, Vascular endothelial growth factor, Tumor microenvironment, Prolyl hydroxylases

## Abstract

Hypoxia, a hallmark of the tumor microenvironment (TME), drives cancer progression through immune modulation, angiogenesis promotion, metabolic reprogramming, and uncontrolled cell proliferation. This review explores the diverse functions of hypoxia-inducible factor (HIF) signaling in cancer development and progression, providing a comprehensive overview of the molecular pathways. HIFs, particularly HIF-1α and HIF-2α, regulate several genes related to cancer hallmarks such as invasion, metabolic reprogramming, angiogenesis, and therapy resistance, thus mediating a significant portion of the hypoxic response. Oxygen-dependent hydroxylation of proline and asparagine residues in HIF-α subunits is a key regulator of their stability and transcriptional activity. Notably, this complex interaction is regulated by multiple signaling pathways, including the extracellular signal-regulated kinase/mitogen-activated protein kinase (ERK/MAPK), phosphoinositide 3-kinase/protein kinase B/mechanistic target of rapamycin (PI3K/Akt/mTOR), and Janus kinase/signal transducer and activator of transcription (JAK/STAT) pathways. In cancer, HIF signaling affects several aspects of tumor cell biology that contribute to the cancerous characteristics, including angiogenesis induction through the upregulation of vascular endothelial growth factor (VEGF) expression, metabolic reprogramming through the enhancement of the Warburg effect, facilitation of cancer invasion and metastasis by driving epithelial-to-mesenchymal transition (EMT) and matrix remodeling patterns, and mediation of therapeutic resistance partly due to the effects on drug efflux pumps and DNA damage repair. Direct and indirect HIF inhibitors—including small molecules, peptidomimetics, antibodies, and proteolysis-targeting chimeras (PROTACs)—are under preclinical and clinical evaluation for their therapeutic efficacy. Preclinical and early clinical trials have demonstrated significant synergistic effects in inhibiting tumor development when HIF inhibition is combined with traditional therapies (chemotherapy or radiation) or immunotherapies, emphasizing major clinical implications and the potential for improving patient outcomes. Although challenges exist, particularly regarding drug resistance, further research to improve therapeutic efficacy and prolong survival for patients is warranted.

## Highlights

• Hypoxia-inducible factors (HIFs) regulate genes involved in angiogenesis, invasion and metabolic reprogramming, essential for cancer progression.
• HIF-1α triggers metabolic changes, including the Warburg effect, to promote tumor survival.
• HIF signaling enhances resistance to chemotherapy and radiation via drug efflux pumps and accelerates DNA repair.
• Natural and synthetic HIF inhibitors, including BAY 87–2243 and glyceollin, are being studied for cancer treatment.
• The combination of HIF inhibitors and conventional therapies demonstrates potential for enhancing cancer treatment results.

## Graphical abstract

**Figure:**
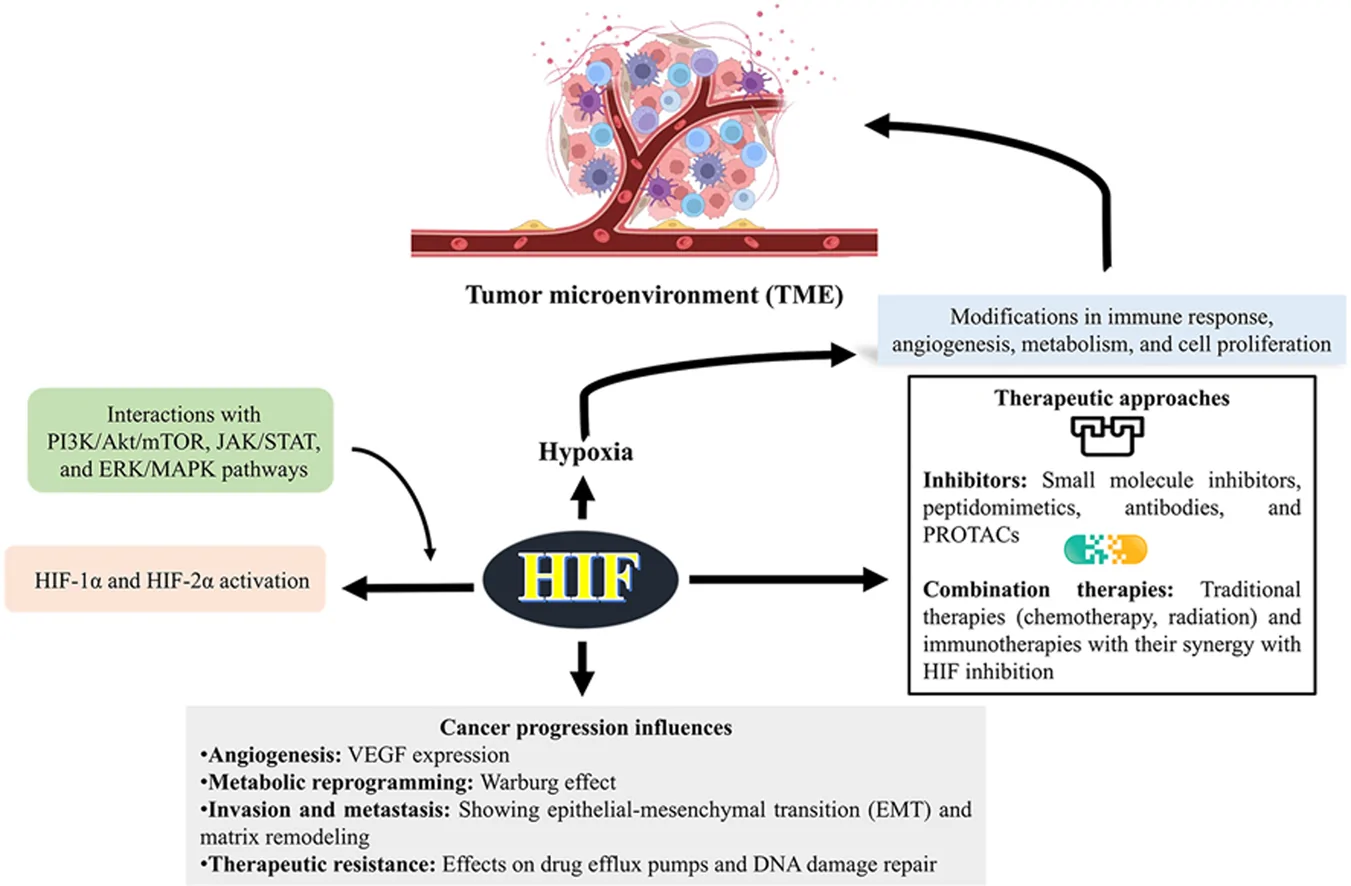

## Introduction

Hypoxia is a crucial component of the tumor microenvironment (TME), and is strongly associated with cell proliferation, metabolism, angiogenesis, and tumor immune response. Hypoxic tissues are those in which oxygen tension <10 mmHg, which is significantly lower than the 40–60 mmHg range observed in most normal tissues.^1^^,^^2^ Accelerated tumor cell proliferation and irregular tumor vasculature development are the direct causes of the start of hypoxia.^3^ The duration of exposure affects how tumor cells react to hypoxia; thus, they are more aggressive during cycles of hypoxia and reoxygenation, leading to necrosis.^4^ The TME can become hypoxic through several mechanisms. A condition called perfusion-restricted hypoxia, sometimes referred to as acute, irregular, temporary, or cyclic hypoxia, occurs when anomalous blood vessels repeatedly close and reopen, resulting in variations in the oxygen supply. These continuous fluctuations can create an uneven distribution of tumor cells, resulting in periodic hypoxia-reoxygenation episodes.^5^

HIF-1, HIF-2, and HIF-3 are some of the heterodimers that make up the family of hypoxia-inducible factors (HIFs), which regulate different hypoxic effects.^6^ Hypoxia stimulates the production of the human erythropoietin (*EPO*) gene by human epithelial protein 3B (Hep3B) cells. Hep3B cells activate the nuclear factor HIF-1 in hypoxic environments, discovered by Gregg Leonard Semenza and his postdoctoral fellow Guang Liang Wang in 1992.^7^ HIF-1 triggers the activation of hypoxic transcription by attaching itself to a particular spot in the *EPO* gene enhancer.^8^

Each of the HIF-1 subunits, HIF-1α and HIF-1β, has a unique function in regulating genes in hypoxic cells.^9^ In normoxic conditions, the HIF-1α subunit is hydroxylated by the prolyl hydroxylase domain (PHD), and HIF-1α is a necessary part of HIF-1. Once the tumor-suppressor E3 ligase von Hippel-Lindau (VHL) protein recognises the hydroxylated HIF component, the proteasome pathway targets it for destruction.^10^ Furthermore, HIF-2 consists of the HIF-2α and HIF-1β subunits, is expressed by certain cell types in vertebrate species. Oxygen controls HIF activity primarily through the hydroxylation of proline and asparagine.^11^

Cancer cells in the TME undergo apoptosis due to deoxygenation, reducing the oxygen levels in particular necrotic areas.^12^ HIF-1α modulates cancer cells, responses and stimulates the expression of several genes linked to the growth and spread of cancer under extreme hypoxic conditions.^11^ Several important features of cancer biology, including angiogenesis,^12^ invasion,^13^ metastasis,^14^ metabolic reprogramming,^15^ signaling of autocrine growth factor,^16^ and defense against radiation therapy and chemotherapy, are facilitated by HIFs.

According to several studies, HIF-1α expression is elevated in several cancers and contributes to disease progression. HIF-1 favorably regulates angiogenesis in cancers and expresses a number of genes linked to cancer stem cell maintenance, metastasis, angiogenesis, cancer metabolism, and resistance to various cancer treatment methods.^17^ In cancer, genes upregulated by HIF-1α including matrix metalloproteinases (MMPs), vascular endothelial growth factor (VEGF),^18^ P53 (*TP53*) and P21 (*CDKN1A*), insulin-like growth factor-2 (*IGF2*), lactate dehydrogenase-A (*LDHA*), nitric oxide synthase (*NOS2*), and transforming growth factor beta 1 (*TGFB1*).^19^

The tumor-suppressive role of HIF-1α has been identified across multiple cancer types. For example, in acute myeloid leukaemia, HIF-1α inhibits the development of the tumor. Increased tumor initiation and progression have been linked to the loss of HIF-1α, which promotes the activation of haematopoietic stem cells (HSCs) and leukemia-initiating cells (LICs).^19^ Recent data indicates that both autophagy and HIF-1α signaling exhibit context-dependent roles in cancer progression, acting as tumor suppressors early on and promoting survival under hypoxia.^20^ HIF-1 induces pluripotent cancer stem cells, promotes selective mitochondrial autophagy, protects cancer cells from T cell-mediated lysis, and aids in tumor development and progression by promoting epithelial-mesenchymal transition (EMT) transitions and E-cadherin loss. Moreover, HIF-1 triggers carcinoma-associated fibroblasts to produce cytokines and signal molecules and upregulate certain microRNAs that are crucial for carcinogenesis.^21^ This review attempts to investigate the diverse functions of HIF signaling in tumor biology, emphasizing on the molecular mechanisms along with roles in regulating cancer-related techniques, including metabolic reprogramming, immune modulation, angiogenesis, invasion, and therapy resistance, while also highlighting emerging therapeutic strategies targeting the HIF pathway.

## Molecular mechanisms underlying hypoxia-inducible factor signaling

### Hypoxia-inducible factor structure and isoforms

HIF is a heterodimeric DNA-binding complex consisting of two basic helix-loop-helix proteins of the Per-ARNT-Sim domain (PAS) family^9^: the oxygen-regulated α-subunit and the constitutively expressed β-subunit (known as aryl hydrocarbon receptor nuclear translocator). There are three identified α-subunit paralogues (HIF-1α, HIF-2α, and HIF-3α) as well as three β-subunit isoforms known as *ARNT1*, *ARNT2*, and *ARNT3*.^22^ These isoforms and paralogues originate from distinct chromosomal regions, and differential mRNA splicing and alternate regulatory elements are used to create additional variations. While HIF-1α and HIF-2α share similar domain structures and proteolytic regulation, HIF-2α exhibits a more tissue-restricted expression pattern.^23^ The inhibitory PAS domain protein, arising from HIF-3α alternative splicing, has both a PAS domain and a basic helix-loop-helix domain at the N-terminus, but it does not have the TAD.^24^ Under hypoxic conditions, the hypoxia response elements (HREs) of target genes, characterized by a conserved core pentanucleotide sequence (RCGTG), are bound by the HIF-α/β heterodimer.^25^

#### HIF-1α vs. HIF-2α

The structural architecture of HIF-1α and HIF-2α isoforms differs significantly, affecting their functional roles.^26^ HIF-1α and HIF-2α differ primarily in their N-terminal transactivation domains (N-TAD), which enhances the target gene selectivity of HIF-1α and HIF-2α. Homologous to all isoforms, the C-terminal transactivation domain (C-TAD) is another transactivation domain that promotes the expression of its common target genes.^27^ N-TAD's oxygen-dependent degradation domain (ODDD) comprises particular proline residues, including Pro402 and Pro564 in HIF-1α, alongside Pro405 and Pro531 in HIF-2α, that undergo hydroxylation by a unique class of PHDs at normal oxygen concentrations. Furthermore, several common genes are regulated by both isoforms, whereas the glycolytic pathway is preferentially activated by HIF-1α.^28^ Genes linked to tumor formation, cell cycle advancement, and stem cell maintenance, such as the proto-oncogene c-Myc and stem cell factor OCT-3/4, are controlled by HIF-2α. In addition, distinct gene groups, with partial overlap, are regulated by HIF-1α and HIF-2α within a single cell type.^27^ HIF-1α and HIF-2α, encoded by distinct genes (*HIF1A* and *EPAS1*, respectively), translate into proteins of varying lengths (826 and 870 amino acids, respectively).^29^

#### Regulation by oxygen levels

Oxygen levels regulate HIF-α through various enzymatic activities, notably through the hydroxylation of two proline residues Pro402 and Pro564 in human HIF-1α, located within the ODDD of the α-subunit.^30^ PHDs, which belong to the larger family of 2-oxoglutarate-dependent dioxygenases (2-OG DD), exist in three isoforms: PHD1, PHD2, and PHD3. These enzymes depend on oxygen, iron (Fe^2+^), and 2-oxoglutarate as cofactors for catalytic activity; therefore, they serve as markers reflecting oxygen availability, cellular metabolic conditions, and iron presence in the cell.^31^ The *VHL* ubiquitin ligase complex binds to HIF-α and catalyzes its polyubiquitination, targeting HIF-α for proteasomal-mediated destruction, and this process is facilitated by the PHD-dependent hydroxylation of HIF-α.^32^ Moreover, HIF-α also experiences oxygen-sensitive hydroxylation, which affects how it binds to the *VHL* tumor suppressor protein (pVHL), a crucial recognition unit of the E3 ubiquitin ligase complex that triggers the ubiquitin-proteasome system to break down HIF-α.^33^

### Transcriptional activity

Proline modification in the ODDD controls HIF-α stability, while the C-terminal asparagine residue (Asn 803 in human HIF-1α) hydroxylation controls transcriptional activity.^34^ In normoxic conditions, asparagine-803 of HIF-1α within the C-TAD is hydroxylated by the factor inhibiting HIF-1 (*FIH-1*), preventing the interaction of HIF-1α with coactivators. Similar to prolyl hydroxylase, *FIH-1* is a dioxygenase enzyme that depends on Fe (II) and 2-oxoglutarate; in addition, it requires vitamin C to keep iron in its ferrous form.^35^ Acting as a secondary oxygen sensor, *FIH-1* uses oxygen as a substrate. Furthermore, pVHL attracts histone deacetylase (HDAC), which impairs transactivation domain function, and the interaction between *FIH-1* and pVHL occurs regardless of *FIH-1*'s enzymatic (hydroxylase) function. Furthermore, the interaction between HIF-1α′s C-TAD and coactivators such as CBP/p300 occurs exclusively under hypoxic conditions, which is controlled by a hydroxylation-dependent switch.^36^ Coactivator proteins promote transcription by separating DNA-binding processes such as adenosine triphosphate (ATP)-dependent chromatin structural remodeling.^37^

#### Coactivators and target genes

Under normoxic conditions, PHD 1–3 must hydroxylate proline (P) residues 402 and 564 in HIF-1α in an oxygen-dependent manner in order to promote the binding of pVHL, an E3 ubiquitin-protein ligase's recognition element, to HIF-1α.^33^ *FIH-1* hydroxylates the asparagine (N) residue 803 of HIF-1α in an oxygen-dependent manner, which prevents HIF-1-driven gene transcription. The binding of p300 and CREB-binding protein (CBP) to HIF-1α is inhibited by this hydroxylation.^35^ Proline and asparagine hydroxylation rates reduce in hypoxic environments. The inability of *VHL* to attach to prolyl-hydroxylated HIF-1α reduces the rate of HIF-1α breakdown.^38^ *FIH-1* inhibits HIF activation by further hydroxylating an asparaginyl moiety at the C-terminus of the HIF-1α and HIF-2α subunits, thereby preventing the binding of coactivators like p300 and its paralog CBP both of which function as histone acetyltransferases to the same region.^39^ In the absence of asparaginyl hydroxylation, HIF-1α can interact with the coactivators p300 and CBP, allowing HIF-1 target genes such as VEGF to be transcriptionally activated, which in turn attracts endothelial cells to hypoxic and avascular regions and promotes their growth.^40^ VEGF, the most effective endothelial cell-specific mitogen, plays a direct role in angiogenesis. This growth factor increases endothelial cell proliferation by interacting with its receptor, the vascular endothelial growth factor receptor (VEGFR), which is primarily expressed in endothelial cells.^41^

Growth factors generated by hypoxia enhance cell survival and proliferation. HIF-1 targets genes associated with many growth factors, including transforming growth factor-α (*TGFA*) and *IGF2*.^42^ The interaction of these molecules with their particular receptors, such as the epidermal growth factor receptor (*EGFR*) and the insulin-like growth factor 1 receptor, starts signal transduction pathways that result in the synthesis of HIF-1α and either supports cell survival or proliferation.^43^

Furthermore, in hypoxic conditions, some cell types exhibit enhanced PI3K activity. The phosphatase and tensin homolog (*PTEN*) inhibits the PI3K pathway, whereas *PTEN* mutations improve HIF-1 activated responses.^44^ In hypoxic environments, cells transition to an oxygen-independent metabolic pathway, with glycolysis serving as the main source of ATP.^45^ Glycolysis generates two ATP molecules for each glucose molecule, whereas the tricarboxylic acid cycle (TCA) generates 38 ATP molecules. Numerous genes related to glycolysis and glucose absorption are HIF-1 target genes.^46^ Besides, the expression of glucose transporter 1 (GLUT1) and glucose transporter 3 (GLUT3) promotes the uptake of glucose by cells, while HIF-1 regulates other enzymes in the glycolytic pathway.^47^ In addition, increased transferrin expression was observed in hypoxic environments, possibly due to improved iron delivery to erythroid tissues. Cellular transferrin absorption is facilitated by the transferrin receptor, a hypoxia-inducible HIF-1 target gene.^48^

**Figure 1:**
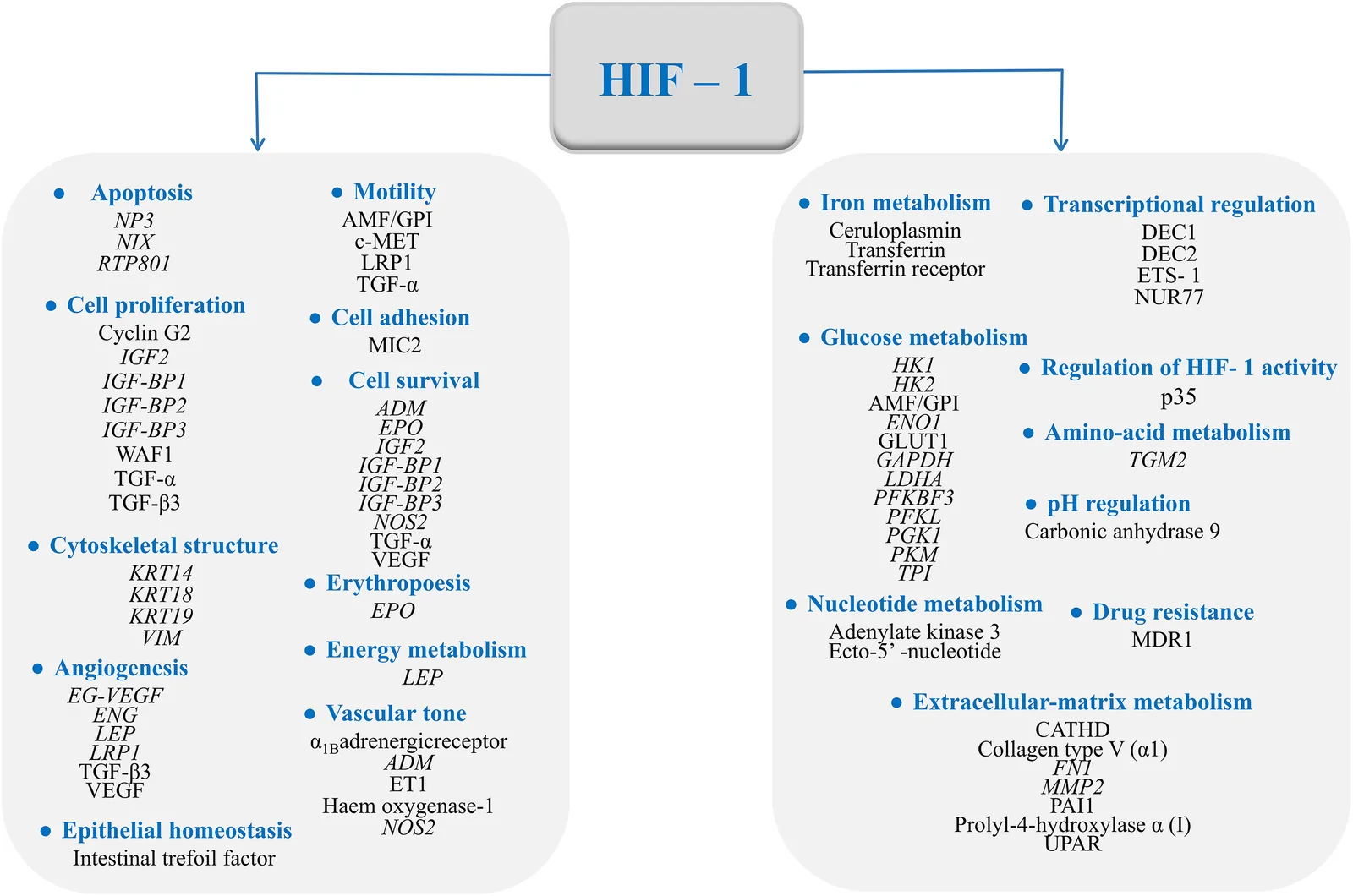
HIF-1-binding genes that trigger transcription. HIF-1-activated genes participate in a number of processes, including *AMF*, *ALDA, CATHD*, *ADM,* EG-VEGF, *ENG*, *ALDC*, ET1, *EPO*, *FN1*, *ENO1,* GLUT1, GLUT3, *GAPDH*, *HK1, HK2*, *IGF2*, *IGF-BP1, IGF-BP2, IGF-BP3*, *KRT14, KRT18, KRT19, LEP*, *LRP1*, MDR1, *MMP-2*, *NOS2, PFKBF3, PFKL, PGK 1,* PAI1, PKM, TGF-α, TGF-β3, VEGF, *TPI*, VEGFR2, UPAR, and *VIM. ADM*: Adrenomedullin; *ALDA:* Aldolase A; *ALDC*: Aldolase C; *AMF*: Autocrine motility factor; *AMF/GP1*: Autocrine motility factor/Glycolytic protein 1; CATHD: Cathepsin D; c-MET: MET proto-oncogene, receptor tyrosine kinase; Cyclin G2: Cyclin G2 phase; DEC1: Differentiated embryo chondrocyte 1; DEC2: Differentiated embryo chondrocyte 2; EG-VEGF: Endocrine-gland-derived VEGF; *ENG*: Endoglin; *ENO1*: Enolase 1; *EPO*: Erythropoietin; ET1: Endothelin-1; *ETS-1*: ETS proto-oncogene 1, transcription factor; *FN1*: Fibronectin 1; *GAPDH:* Glyceraldehyde-3-P-dehydrogenase; GLUT1: Glucose transporter 1; GLUT3: Glucose transporter 3; HIF-1: Hypoxia-inducible factor 1; *HK1*: Hexokinase 1; *HK2*: Hexokinase 2; *IGF-BP1*: Insulin-like growth factor-binding protein 1; *IGF-BP2*: Insulin-like growth factor-binding protein 2; *IGF-BP3*: Insulin-like growth factor-binding protein 3; *IGF2*: Insulin-like growth factor 2; *KRT14*: Keratins 14*; KRT18*: Keratins 18; *KRT19*: Keratins 19; *LDHA*: Lactate dehydrogenase A; *LEP*: Leptin; *LRP1*: Receptor-related protein 1; MDR1: Multidrug resistance 1; MIC2: Membrane glycoprotein MIC2; *MMP-2*: Matrix metalloproteinase-2; NIX: NIP3-like protein X; *NOS2*: Nitric oxide synthase 2; NP3: Neuropeptide Y receptor type 3; NUR77: Nuclear receptor subfamily 4 group A member 1; PAI1: Plasminogen-activator inhibitor 1; *PFKBF3*: 6-phosphofructo-2-kinase/fructose-2,6-biphosphatase-3; *PFKL*: Phosphofructokinase L; *PGK 1*: Phosphoglycerate kinase 1; PKM: Pyruvate kinase M; p35: Cyclin-dependent kinase 5 activator 1; RTP801: DNA damage-inducible transcript 4; *TGF-α*: Transforming growth factors α; TGF-β3: Transforming growth factors β3; TPI: Triosephosphate isomerase; UPAR: Urokinase plasminogen activator receptor; VEGF: Vascular endothelial growth factor; VEGFR2: Vascular endothelial growth factor receptor 2; *VIM*: Vimentin; WAF1: Wild-type p53-activated fragment 1.

Furthermore, the gene encoding ceruloplasmin (*CP*) has been identified as a downstream target of *HIF-1*. *CP*, also referred to ferroxidase, is necessary for the conversion of ferrous to ferric iron. Since transferrin is the only protein that binds ferric iron, hypoxic CP induction should increase the amount of iron that reaches erythroid tissue.^46^ Figure 1 illustrates that several genes associated with angiogenesis, metabolism, cell survival, and invasion are activated by HIF-1, demonstrating its importance in tumor progression.

#### Non-canonical hypoxia-inducible factor signaling

Phosphoinositide 3-OH kinase/protein kinase B/mechanistic target of rapamycin (PI3K/Akt/mTOR), Extracellular signal-regulated kinase/mitogen-activated protein kinase (ERK/MAPK), and Janus kinase/signal transducer and activator of transcription (JAK/STAT) are the three main pro-survival pathways that synergistically promote HIF-1α transcription and translation, particularly in cancer.^49^ ERK1/2-mediated MAPK signaling enhances HIF-1 transactivation capacity via phosphorylation-dependent regulation of p300/CBP coactivators complex.^50^ Through signal transducer and activator of transcription 3 (STAT3) activation, JAK/STAT signaling triggers an Akt-mediated pathway that enhances HIF-1α gene expression,^51^ and the PI3K/Akt/mTOR pathway directly upregulates HIF-1α expression through dual control of both its transcriptional activation and translational synthesis.^52^ Deficiencies in oxidative phosphorylation lead to elevated reactive oxygen species (ROS) generation. In addition, various conditions such as accumulation of ROS and reactive nitrogen species induced by mitochondrial DNA (mtDNA) mutations, intermittent hypoxia, inflammatory mediators, and chemical toxicants can activate the PI3K/Akt/mTOR pathway, leading to enhanced HIF-1α expression through both transcriptional and translational upregulation.^53^ Furthermore, through ERK-PI3K/Akt signaling, cells upregulate HSP90 chaperone proteins that stabilize HIF-1α without needing hypoxia. At the same time, the energy-sensing 5′-adenosine monophosphate kinase (AMPK) protein puts the brakes on this system by blocking mTOR activation.^54^ HIF-1α degradation may be inhibited by oxidative stress-induced AMPK activation, and activated AMPK promotes ROS-induced HIF-1α upregulation.^55^

## Impact of hypoxia-inducible factor signaling in the progression of cancer

Table 1 presents that HIF signaling affects various cancer types through the stimulation of blood vessel growth, cell division, and metastasis, which ultimately leads to poorer patient outcomes.

**Table 1:** Roles of hypoxia-inducible factor signaling in different cancer types.

Site	Mechanism	Effect	Reference
Breast	Upregulates VEGF expression, blood vessel growth, and cancer cell dormancy	Promotes malignant spread and osteolytic bone metastases	165
Lung	Overexpression of VEGF causes ROS generation.	Increases lymphatic infiltration, minimal tumor differentiation, Enhanced multiplication, and unfavorable outlook	166
Bone	Prevention of mesenchymal cell development into osteoblasts and promotion of osteoclast differentiation by increasing IGF, VEGF, and RANKL levels	Osteolysis, osteoblast apoptosis, suppression of expression of osteocalcin	167
Colorectal	Expression of the ZEB gene, elevated the synthesis of VEGF, COX2, and MMP	Elevates cancer cell proliferation and dissemination, as well as the EMT process	168
Prostate	Increases expression of growth factors and enzymes	Stress-induced hyperproliferation	169

COX2: Cyclooxygenase-2; EMT: Epithelial-mesenchymal transition; HIF: Hypoxia-inducible factor; IGF: Insulin-like growth factor; MMP: Matrix metalloproteinase; ROS: Reactive oxygen species; RANKL: Receptor activator of nuclear factor kappa-B ligand; VEGF: Vascular endothelial growth factor; ZEB: Zinc finger E-box binding homeobox.

**Figure 2:**
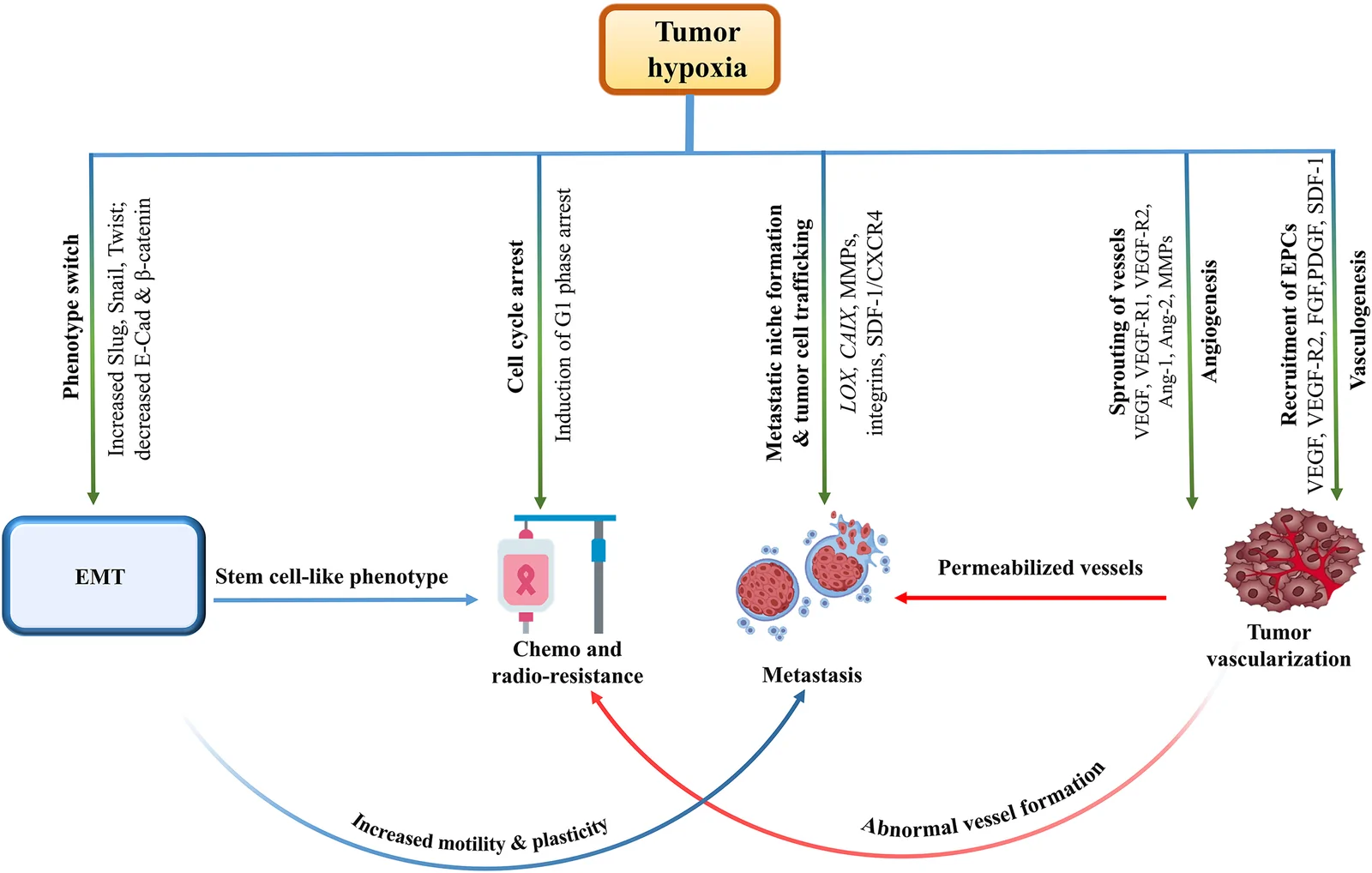
Hypoxia promotes tumor vasculogenesis by mobilizing endothelial progenitor cells, a process affected by GF and their receptors. Hypoxia stimulates angiogenesis through pre-existing vessel sprouting, mediated by increased GF, MMPs, Ang-1, and Ang-2. New blood vessels aid cancer cell migration, enhanced by *LOX*, CAX, MMPs, integrins, and *CXCR4*. Hypoxic cancer cells undergo EMT, gaining plasticity and mobility through transcription factors (Slug, Snail, Twist) and decreased adhesion molecules (β-catenin, E-cadherin). Chemoresistance arises from EMT-related stemness and hypoxia-induced G1 phase cell cycle arrest. Abnormal vascularity further hinders drug diffusion. Ang-1: Angiopoietin-1; Ang-2: Angiopoietin-2; CAX: Carbonic anhydrase X; CAIX: Carbonic anhydrase IX; EMT: Epithelial-mesenchymal transition; FGF: Fibroblast growth factors; G1: G1 phase of the cell cycle; GF: Growth factors; *LOX*: Lysyl oxidase; MMPs: Matrix metalloproteinases; PDGF: Platelet-derived growth factor; SDF-1: Stromal cell-derived factor 1; SDF-1/CXCR4: Stromal cell-derived factor 1/C-X-C chemokine receptor type 4; Slug (Snai2): Snail family transcriptional repressor 2; SNAIL (Snai1): Snail family transcriptional repressor 1; TWIST: Twist-related protein 1; VEGF: Vascular endothelial growth factor; VEGF-R1: Vascular endothelial growth factor receptor 1; VEGF-R2: Vascular endothelial growth factor receptor 2.

By changing the TME, hypoxia enhances vasculogenesis and angiogenesis, facilitates cancer cell migration, and triggers chemoresistance [Figure 2].

### Angiogenesis

#### Vascular endothelial growth factor and angiogenic switch

Angiogenesis—formation of new blood vessels from pre-existing vasculature—is regulated by the angiogenic switch, which balances pro-angiogenic and anti-angiogenic molecules in cells. Under hypoxic conditions, HIF-1α stabilizes and activates several genes which break the angiogenic shift, thereby inducing and increasing the rate of angiogenesis.^56^ Pro-angiogenic signaling is triggered by pathophysiological stressors such as hypoxia, leading to higher tissue bulk, vascular dysfunction, and vessel occlusion.^57^ Several pro-angiogenic factors, including VEGF, plasminogen activator inhibitor-1, VEGF receptors *FLT-1* and FLK-1, platelet-derived growth factor B (*PDGF-B*), stromal-derived factor 1a (SDF-1a), and stem cell factor (SCF), angiopoietins (ANG-1 and ANG-2), and MMPs (*MMP-2* and *MMP-9*), are stimulated by HIF.^58^ Hypoxia causes the expression of VEGF messenger RNA (mRNA) and protein, indicating that it promotes angiogenesis by upregulating VEGF expression. As a result, placenta growth factor/VEGF heterodimers are formed, and when co-expressed, they alter VEGF activity.^59^ *IGF2* increases VEGF secretion, which contributes to the angiogenic stage in hepatocellular carcinoma. In addition, *IGF2* considerably boosts the levels of VEGF mRNA and protein in HepG2 cells and enhances HIF-1 protein stabilization, which plays a crucial factor in the induction of VEGF expression.^60^ Table 2 lists different pro-angiogenic and anti-angiogenic factors.

**Table 2:** HIF-induced anti-and pro-angiogenic factors.^170^

Anti-angiogenic factors	Pro-angiogenic factors
CA-9	ADM
Regulator of G protein signaling 5	Flt-1 (VEGF-R1), Kdr (VEGF-R2), VEGF
DLL1-4	FGF
Angiostatin	PLGF
Canstatin	Tie-2, Ang-1/2
Vasohibin-1	PDGF-B
Endostatin	SCF
Thrombospondin-1	Osteopontin
	Beta-1-adrenergic receptor
	NOS
	Interleukins (IL 1–6, IL-8, IL-10)
	Endoglin
	MMP, TIMP
	Integrins, leptin
	Semaphorin 4D
	Endothelin-1
	Endosialin
	SDF-1
	Oxygen-regulated protein–150
	Adenosine A2A receptor
	COX-2

A2A: Adenosine A2A receptor; ADM: Adrenomedullin; Ang-1/2: Angiopoietin-1/Angiopoietin-2; CA-9: Carbonic anhydrase-9; COX-2: Cyclooxygenase-2; DLL1-4: Delta-like ligand 1–4 (Notch signaling pathway ligands); FGF: Fibroblast growth factors; HIF: Hypoxia-inducible factor; IL: Interleukins; MMP: Matrix metalloproteinase; NOS: Nitric oxide synthase; PDGF-B: Platelet-derived growth factor B; PLGF: Placental growth factor; SCF: Stem cell factor; SDF-1: Stromal-derived factor 1; TIE2: Tyrosine kinase with immunoglobulin-like and EGF-like domains 2; TIMP: Tissue inhibitor of metalloproteinases; VEGF: Vascular endothelial growth factor; VEGF-R1/2: Vascular endothelial growth factor receptor 1/2.

#### Effect of angiogenesis on tumor vascular architecture

Angiogenic switch occurs when tumor growth is inhibited, limited by the oxygen and nutrient supply, necessitating new vessel formation. Pathological angiogenesis, driven by persistent pro-angiogenic signaling, causes the formation of abnormally dilated, leaky tumor blood vessels that boost intratumoral pressure, promote metastasis, and reduce the effectiveness of chemotherapy, and weaken the body's immune response against tumors.^61^ Tumor blood vessels possess structural and functional defects caused by dysregulated angiogenic signaling. Proangiogenic factors from tumor and stromal cells help to sustain tumor-induced angiogenesis.^62^ The VEGF family regulates angiogenesis by encoding ligands that enhance new blood vessels development in normal as well as malignant tissues. Tissue homeostasis and vascular permeability depend on VEGF-A.^63^ Angiogenesis in physiology produces functioning vessels, whereas angiogenesis in malignancies produces dysfunctional vasculature with abnormal functions, which results in insufficient tumor perfusion.^64^ HIF-1-regulated angiopoietin-like 4 and VEGF promote vascular dispersion, leading to increased permeability and microvessel density by disrupting cell junctions.^65^

### Metabolic reprogramming

#### The warburg effect and beyond

**Figure 3:**
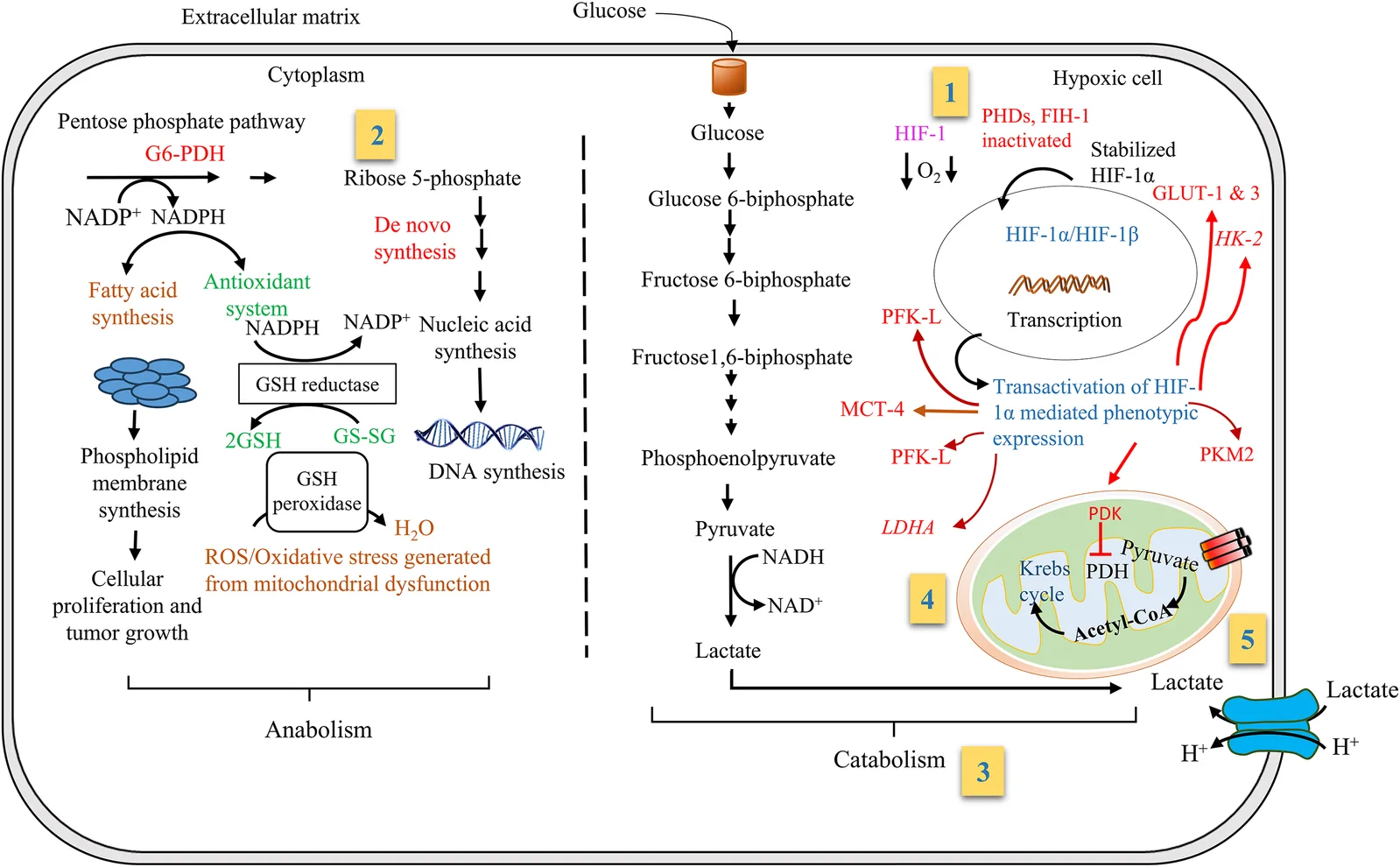
Glucose metabolism in healthy cells primarily occurs through glycolysis, the TCA cycle, and oxidative phosphorylation, leading to ATP production. This process involves the interconversion of isoenzymes such as NADH, FAD, and ADP, creating an electron gradient. In hypoxic conditions, cells shift from oxidative phosphorylation to anaerobic glycolysis due to oxygen deficiency. This causes pyruvate accumulation, which is subsequently reduced to lactate by LDHA. HIF-1α enhances ATP production via upregulation. ADP: Adenosine diphosphate; ATP: Adenosine triphosphate; FAD: Flavin adenine dinucleotide; G6-PDH: Glucose-6-phosphate dehydrogenase; GS-SG: Glutathione disulfide (oxidized glutathione); GSH: Glutathione (reduced form); GLUT-1: Glucose transporters 1; GLUT-3: Glucose transporters 3; HIF: Hypoxia-inducible factor; HIF-1α/β: Hypoxia-inducible factor-1α/β; *HK-2*: Hexokinase 2; LDHA: Lactate dehydrogenase A; MCT-4: Monocarboxylate transporter 4; NAD: Nicotinamide adenine dinucleotide (oxidized form); NADH: Nicotinamide adenine dinucleotide (reduced form); NADP: Nicotinamide adenine dinucleotide phosphate (oxidized form); NADPH: Nicotinamide adenine dinucleotide phosphate (reduced form); PDK: Pyruvate dehydrogenase kinase; PDH: Pyruvate dehydrogenase; PFK-L: Phosphofructokinase L; PHDs: Prolyl hydroxylase domain enzymes; PKM2: Pyruvate kinase M2; TCA: Tricarboxylic acid.

Otto Warburg discovered the Warburg effect in 1924, which describes how cancer cells preferentially use glycolysis to generate ATP, nucleotides, amino acids, and lipids needed for cancer cell proliferation, even in the presence of oxygen (aerobic conditions).^66^ HIF-1α stimulates the Warburg effect by upregulating glucose transporters (GLUT1 and GLUT3) and key glycolytic enzymes including hexokinases (HK1/HK2), phosphoglycerate kinase 1 (PGK-1), and LDHA, playing a crucial role in the shift to anaerobic glycolysis from oxidative phosphorylation.^67^ GLUT transporters enhance glucose uptake into cells, promoting glycolysis. The HIF-1 protein complex penetrates the nucleus, activating transcription of *SLC2A1* and *SLC2A3* genes and subsequent translation, leading to increased expression of GLUT1 and GLUT3 glucose transporters.^68^ HIF-1 activation boosts HK1 and HK2 (key glycolytic enzymes that convert glucose into glucose-6-phosphate), thereby directing glucose toward the glycolytic pathway in tumor cells.^69^ Additionally, HIF-1α activates LDHA to convert pyruvate and NADH into lactate and NAD^+^, a key step in supporting cancer cell metabolism.^70^ In hypoxic conditions, as shown in Figure 3, there is a metabolic reprogramming from oxidative phosphorylation to glycolysis, which is facilitated by HIF-1α activation to ensure the survival of cancer cells.

**Figure 4:**
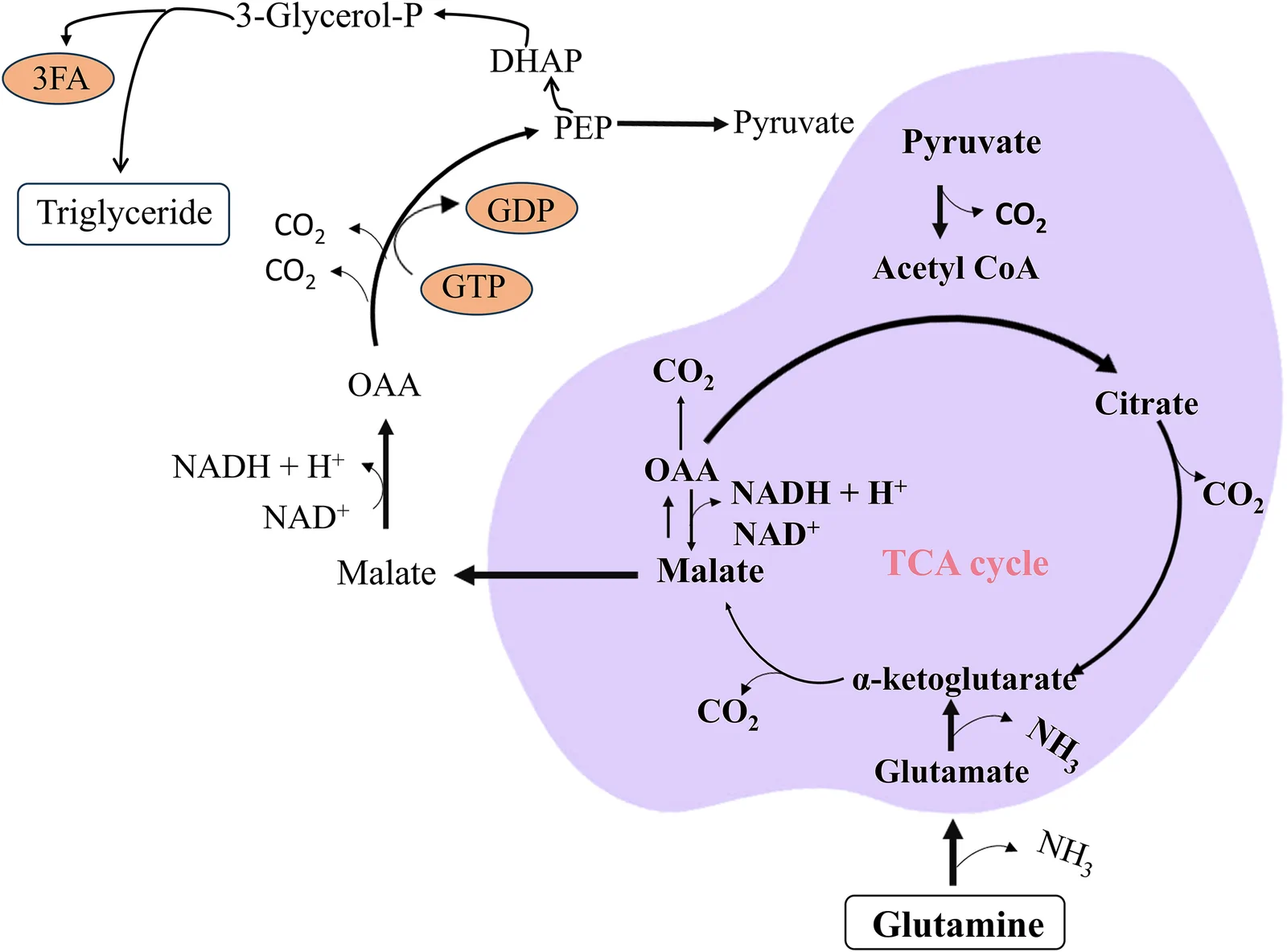
Glutamine metabolism involves anaplerosis (entry into the TCA cycle) and cataplerosis (removal as malate). Malate is converted to OAA and then to PEP via PEPCK. PEP turns into pyruvate, entering the TCA cycle as acetyl-CoA or converting to alanine. Glyceroneogenesis forms 3-Glycerol-P for triglyceride synthesis, balancing OAA entry and intermediate removal. DHAP is reduced to 3-Glycerol-P, providing the glycerol backbone for new triglycerides. Solid arrows indicate anaplerosis pathways; red arrows show glyceroneogenesis pathways; dotted arrow represents mitochondrial cataplerosis. 3FA: 3 Fatty acids; 3-Glycerol-P: l-glycerol-3-phosphate; CoA: Coenzyme A; DHAP: Dihydroxyacetone phosphate; GDP: Guanosine diphosphate; GTP: Guanosine triphosphate; NAD: Nicotinamide adenine dinucleotide (oxidized form); NADH: Nicotinamide adenine dinucleotide (reduced form); OAA: Oxaloacetate; PEP: Phosphoenolpyruvate; PEPCK: Phosphoenolpyruvate carboxykinase; TCA: Tricarboxylic acid.

HIF-1α transcriptionally upregulates monocarboxylate transporter 4 (MCT4), a key plasma membrane protein that converts pyruvate to lactate.^71^ The *MYC* oncogene regulated glutamine uptake and metabolism, potentially affecting *MYC*-transformed cells. Glutamine is crucial for TCA cycle intermediates and biomass production, and it can be metabolized through glutaminolysis, which contributes to glyceroneogenesis.^72^ This interconnected pathway, involving anaplerosis and cataplerosis of TCA intermediates and their diversion into lipid biosynthesis, is illustrated in Figure 4.

#### Mitochondrial function

**Figure 5:**
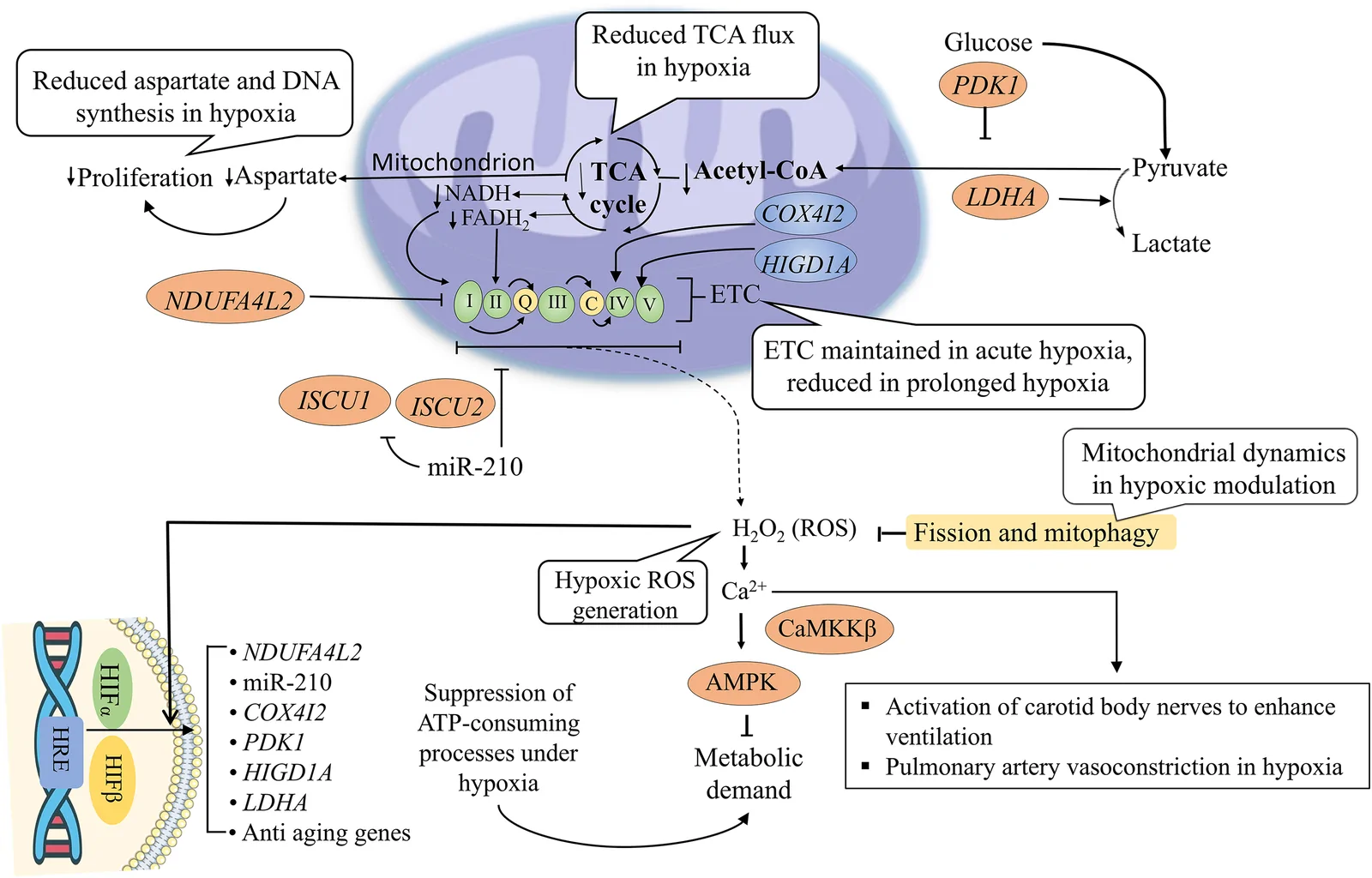
The effects of hypoxia on mitochondrial activity. Hypoxia affects mitochondrial function by reducing flux through the TCA cycle, leading to decreased levels of metabolites such as acetyl-CoA and aspartate, essential for anabolic processes. HIF-1 induces *LDHA* and *PDK1* expression, inhibiting pyruvate entry into the TCA cycle by promoting lactate generation and blocking its conversion to acetyl-CoA. Acute hypoxia maintains ETC activity via *COX4I2* expression, replacing *COX4I1* for efficient electron transfer. *HIGD1A* enhances COX activity. Prolonged hypoxia diminishes ETC activity through N*DUFA4L2* and *miR-210*, reducing complex I activity and assembly, thus lowering ROS production. Hypoxia-induced ROS, particularly H_2_O_2_, signal various cellular responses, including increased Ca^2+^ levels that activate CaMKK, which in turn activate AMPK to suppress ATP-consuming processes. Ca^2+^ signals carotid body nerves and pulmonary artery vasoconstriction. Moderate ROS levels have anti-aging functions. Hypoxia modulates mitochondrial dynamics (fission), enhancing damaged mitochondria quality control via mitophagy, thereby limiting ROS generation. AMPK: 5′-adenosine monophosphate kinase; ATP: Adenosine triphosphate; CaMKK: Calcium/calmodulin-dependent protein kinase; CaMKKβ: Calcium/calmodulin-dependent protein kinase beta (CaMKK2); *COX*: Cytochrome *c* oxidase; *COX4I1*: Cytochrome *c* oxidase subunit 4 isoform 1; CoA: Coenzyme A; *COX4I2*: Cytochrome *c* oxidase subunit 4 isoform 2; ETC: Electron transport chain; FADH: Flavin adenine dinucleotide; HIF: Hypoxia-inducible factor; HIF-1α: Hypoxia-inducible factor-1α; HIF-1β: Hypoxia-inducible factor-1β; *HIGD1A*: HIG1 hypoxia inducible domain family member 1A; HRE: hypoxia response elements; ISCU1: Iron-sulfur cluster assembly enzyme 1; ISCU2: Iron-sulfur cluster assembly enzyme 2; *LDHA*: Lactate dehydrogenase A; *miR-210*: MicroRNA-210; NADH: Nicotinamide adenine dinucleotide (reduced form); *NDUFA4L2*: NADH dehydrogenase [ubiquinone] 1 alpha subcomplex 4-like 2; *PDK1*: Pyruvate dehydrogenase kinase 1; ROS: Reactive oxygen species; TCA: Tricarboxylic acid.

Hypoxia stimulates HIF-1, which shifts cellular metabolism from aerobic respiration toward anaerobic glycolysis by limiting mitochondrial function and altering oxidative phosphorylation, and stimulating gene expression.^73^ HIF-1 reduces mitochondrial function in cancer through several mechanisms, notably hypoxia modifying the expression of cytochrome C oxidase (COX) element, thereby optimizing respiration efficiency at varying oxygen levels.^74^ The TCA cycle is suppressed by HIF-1, and this suppression is caused by the pyruvate dehydrogenase kinase 1 (*PDK1*) gene,^73^ as *PDK1* inhibits the PDH enzyme, which converts pyruvate to acetyl-CoA for TCA cycle entry. Moreover, HIF-1 directly impairs mitochondrial biogenesis and oxidative respiration in *VHL*-deficient renal carcinoma cells by transcriptionally repressing c-Myc.^75^ Hypoxia triggers autophagy, removing damaged organelles like mitochondria and releasing nutrients for cell survival, thereby promoting survival in stressful environments.^76^ In addition, hypoxia increases the position of Akt in mitochondria, with a more intense phosphorylation of *PDK1* on Thr346, thus hindering autophagy.^77^ Autophagy stimulation through *HK2*-mediated repression of TORC1 has been reported in glycose-starved neonatal rat ventricular myocytes.^78^ The various effects of hypoxia on mitochondrial activity, such as reduced TCA cycle flux, changed electron transport chain function, and suppressesed autophagy, are depicted in Figure 5.

### Invasion and metastasis

#### Epithelial-mesenchymaltransition

In EMT, mesenchymal markers such as snail family transcriptional repressor 1 (Snail/Snai1), vimentin, and N-cadherin are upregulated, whereas epithelial markers such as cytokeratins, claudins, desmoplakins, and E-cadherin are downregulated.^79^ Hypoxia-inducible genes, including *SNAI1*, SLUG *(SNAI2),* and TWIST *(TWIST1* and *TWIST2),* regulate EMT by downregulating E-cadherin expression, which is crucial for epithelial cell junctions and structural arrangement of tissue.^80^ EMT target genes such as E-cadherin (*CDH1*), claudins (*CLDN*), occluding (*OCLN*), PALS1 (*MPP5*), and PATJ (*INADL)* are all downregulated by SNAIL1 and SNAIL2.^81^ During hypoxia, TWIST facilitates EMT by not only enhancing HIF-1α protein stability but also stimulating TGF-β1 signaling, creating a synergistic loop that amplifies HIF-1α levels and activates mesenchymal transition.^82^ In head and neck squamous cell carcinomas (HNSCC), the mesenchymal phenotype triggered by hypoxia is largely driven by SLUG, which is regulated by HIF-1α and contributes to the Cadherin switch.^83^

#### Matrix remodeling

**Figure 6:**
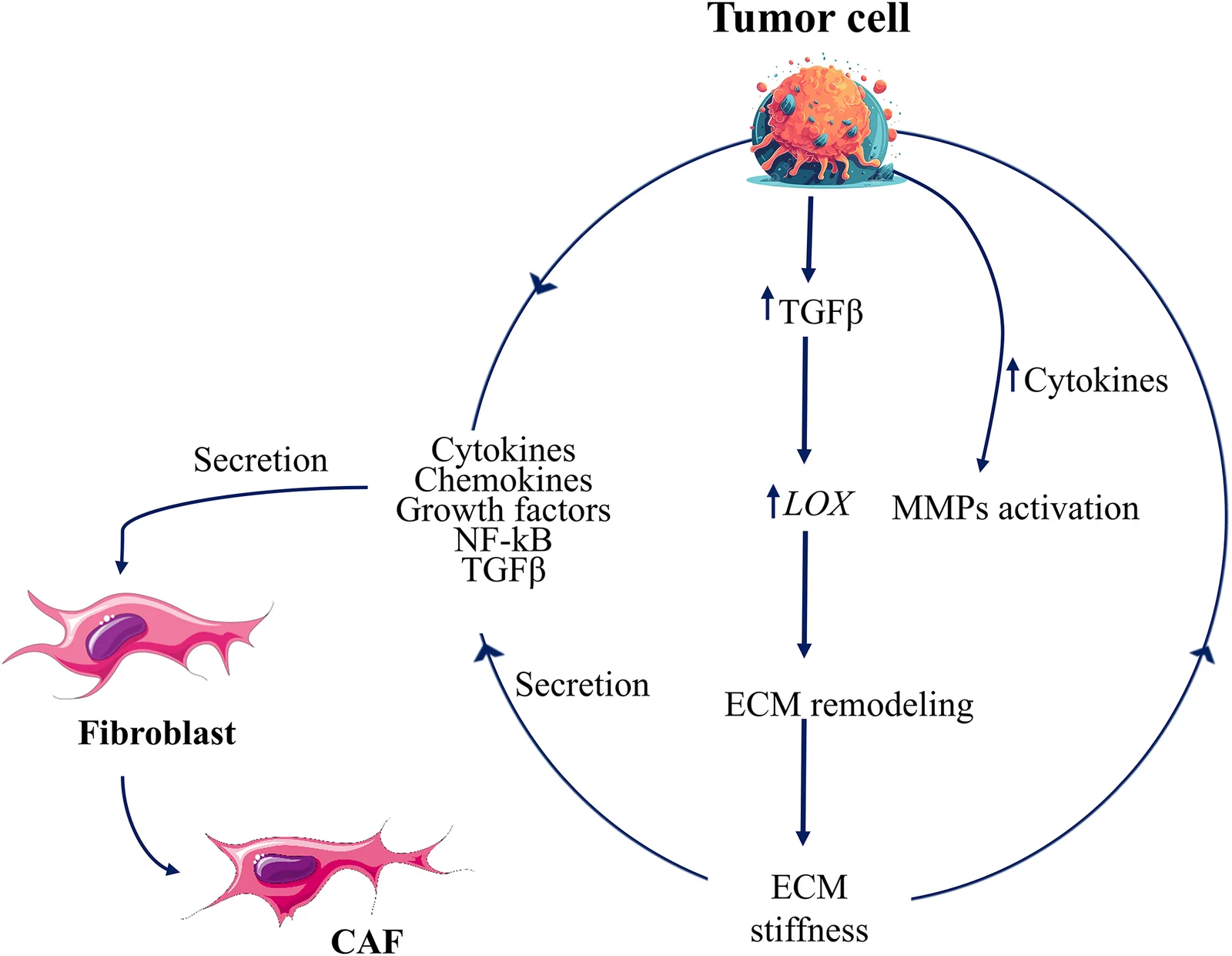
ECM remodeling and cancer cell-stroma cell interactions are mediated by cell signaling mediators. Tumor cells generate cytokines, chemokines, NF-κB, and TGFβ, which “activate” stromal progenitors such as fibroblasts into CAFs. Afterwards, these CAFs produce more cytokines and TGFβ, which affect tumor cell functions. TGFβ overexpression in cancerous cells increases *LOX* level, which causes ECM stiffness and alters collagen organization, affecting ECM remodeling and mechanically activating latent TGFβ. Tumor cells that produce more cytokines activate MMPs, which aid in ECM remodeling and maintain tumor growth. CAF: Cancer-associated fibroblast; ECM: Extracellular matrix; *LOX*: Lysyl oxidase; MMPs: Matrix metalloproteinases; NF-κB: Nuclear factor kappa-light-chain-enhancer of activated B cells; TGFβ: Transforming growth factor beta.

The extracellular matrix (ECM), comprising glycoproteins, collagen, elastin fibrils, proteases, glycosaminoglycans, and proteoglycans, serves as a regulatory environment for tumor cells, promoting proliferation, migration, adhesion, invasion, and metastasis.^84^ MMPs are zinc-dependent endopeptidases that target ECM proteins and promote connective tissue remodeling.^85^ HIF-1α remodels collagen in the ECM and promotes fibrosis. In solid tumors, hypoxia-induced activity enhances tumor development and metastasis by modifying the ECM. Furthermore, it enhances collagen production and boosts fibrotic ECM conditions, activating MMPs including *MMP-2, MMP-9*, and *MMP-15*. They are responsible for ECM breakdown and remodeling, which can lead to invasion and metastasis^86^^,^^87^; for example, *MMP-2* enhances squamous cell carcinoma metastasis via EMT, potentially affecting patient prognosis.^88^ Thus, ECM remodeling driven by HIF-1α plays a vital role in shaping the TME, making it more susceptible to tumor growth, invasion and metastasis,^89^ as illustrated in Figure 6.

#### Pre-metastatic niche

A pre-metastatic niche (PMN) is a specialized microenvironment at future metastatic sites that supports cancer cell attachment, survival, colonization, and propagation. Soluble factors secreted by primary tumors influence hematopoietic bone marrow-derived cells (BMDCs) to create the pre-metastatic niche (PMN), which facilitates metastatic seeding in distant organs.^90^ Targeting hypoxia, LOX attracts CD11b^+^ myeloid cells under hypoxia to promote the colonizing of metastatic tumor cells by forming a niche.^91^ The lung is a common metastatic site, where primary cancer cells modify the ECM, resulting in the accumulation of BMDCs, thus establishing an environment rich in growth factors for the colonization of cancer cells.^92^ In addition, primary cancer cells promote metastasis by producing more chemokines like SDF-1, which recruit CXCR4^+^ tumor cells to sites of metastasis^92^; this process generates neovessels in the lesion and suppresses immune responses mediated by natural killer cells via CD11b^+^ BMDCs, thereby encouraging cancer cell retention in tumor-bearing animals.^93^ The liver is frequently affected by metastases from colorectal and pancreatic cancers; although many pathogens pass through the liver, it is protected by Kupffer cells. These cells induce TGF-β, fibronectin release in hepatic stellate cells and recruit tumor-associated macrophages to the liver.^94^

### Therapeutic resistance

#### Chemotherapy and radiotherapy resistance

Efflux pumps are common mechanisms in cancer cells that actively expel chemotherapy drugs, thereby reducing their intracellular concentration and efficacy.^95^ Drug efflux routes such as MDR1 (P-glycoprotein/ATP-binding cassette B1 [*ABCB1*]), MRP1 (multidrug resistance-associated protein 1/*ABCC1*) as well as breast cancer resistance protein (BCRP/*ABCG2*) are crucial in cancer resistance. These ATP-binding cassette (ABC) protein transporters mediate active efflux of chemotherapeutic agents across cellular membranes, representing a primary mechanism of multidrug resistance in cancer cells.^86^ MRP1 is linked to HIF-1-mediated drug resistance that occurs in hypoxic conditions.^96^ Drug resistance in leukemia and breast cancer is linked to BCRP, and cancer stem cells exhibit significant levels of MDR transporter upregulation, specifically for BCRP.^97^ The DNA-damage response (DDR) approach detects DNA defect and preserves genome integrity. Mutations in DDR genes lead to genomic instability and increase repair frequency during cancer progression.^98^ Table 3 summarizes numerous HIF-mediated pathways contributing to drug resistance, detailing specific cancer models, the resistance phenotype, and the genetic basis underlying these adaptations.

**Table 3:** A summary of drug resistance pathways mediated by HIF.

Molecule/drug	Resistance phenotype	(Cancer) Cell model	Molecular origin	Reference
Adriamycin	Drug efflux	Glioblastoma cells, colon cancer cells	P-gp	171
Etoposide, doxorubicin	Drug efflux	Glioma cells	MRP1	172
5-Fluorouracil, cisplatin	Drug efflux	OSCC cells	P-gp	173
Multiple drugs	Drug efflux	Gastric cancer cells	P-gp, MRP1	174
5-Fluorouracil	Drug efflux	HCC cells	P-gp, MRP1, LRP	96
Methotrexate	Drug efflux	Breast cancer cells	P-gp	175
Etoposide	DNA damage inhibition	Mouse embryonic fibroblasts	DNA-dependent protein kinase complex	176
Etoposide, carboplatin		Mouse embryonic fibroblasts		177
Etoposide	DNA damage inhibition	Breast cancer cells, prostate cancer cells	Topoisomerase II alpha	178

HCC: Hepatocellular carcinoma; HIF: Hypoxia-inducible factor; LRP: Lung resistance-related protein; MRP1: MDR-associated protein 1/*ABCC1*; OSCC: Oral squamous cell carcinoma; P-gp: P-glycoprotein.

#### Targeted therapy resistance

Key pro-angiogenic factors and their receptors expressed on endothelial cells such as VEGF, VEGFR1, VEGFR2, VEGFR3, TIE2, ANG1, FGFR, FGF2, HGF, and c-Met are typically targeted by anti-angiogenic drugs.^99^ Anti-angiogenic drugs reduce tumor development and metastasis by reducing blood flow to tumor tissue, resulting in hypoxia, which may explain the currently available drugs with inadequate therapeutic efficacy due to evasive resistance mechanisms.^62^ Furthermore, anti-angiogenic resistance may arise when angiogenic genes are overexpressed, angiogenic factors are secreted more, and angiogenic bone marrow-derived cells are recruited in greater numbers.^100^ Notch signaling inhibits VEGF signaling, resulting in resistance.^101^ In addition, adaptive resistance occurs when therapy changes the tumor and its vasculature owing to factors such as tumor expression, metabolic reprogramming, hypoxia-induced invasion, upregulation of pro-angiogenic proteins, and greater recruitment of protective pericyte coverage.^102^ Hypoxia-mediated chemoresistance to medications such as doxorubicin, cisplatin, and etoposide is observed in neuroblastoma,^103^ whereas cisplatin and 5-fluorouracil resistance is acquired by HNSCC and gastric cancer.^104^

## Therapeutic targeting of hypoxia-inducible factor signaling

There are two categories of HIF inhibitors: (1) direct HIF inhibitors function by altering the activity of HIF molecules themselves, (2) indirect inhibitors exert their effects by targeting regulatory components in upstream or downstream pathways, thereby influencing the overall HIF signaling cascade.^105^

### Direct inhibitors of hypoxia-inducible factor

#### Small molecule inhibitors

BAY 87–2243 effectively reduces HIF-1 expression in head and neck cancer xenografts, which enhances local tumor control and provides a basis for further clinical trials.^106^ PT-2399 (2,3-dihydro-1H-inden-4-yl-oxy derivative) and PT-2385 exhibit anti-angiogenic actions in xenograft models of *VHL*-deficient clear cell renal cell carcinoma (ccRCC) by blocking HIF-2α-ARNT heterodimer formation.^107^ DJ12 reduces VEGF formation in melanoma, breast, and kidney cancer cell lines by inhibiting HIF-1α′s capacity to bind to DNA. Acriflavine, rolitetracycline, and THS-044 interfere with transcriptional activity by binding to the PAS domain and blocking the dimerization of HIF-α with HIF-β.^108^ Furthermore, YC-1 not only increases HIF-1α degradation but also inhibits HIF-1α production and impairs its transcription ability by preventing HIF-1α-p300 interaction.^109^ PX-478 suppresses HIF-1α by decreasing mRNA and protein expression and inhibiting both its transactivation and translation processes.^110^ Camptothecin, the first identified HIF-1 inhibitor, works by preventing HIF-1 activation, which suppresses protein accumulation.^111^

#### Peptidomimetics and antibodies

**Figure 7:**
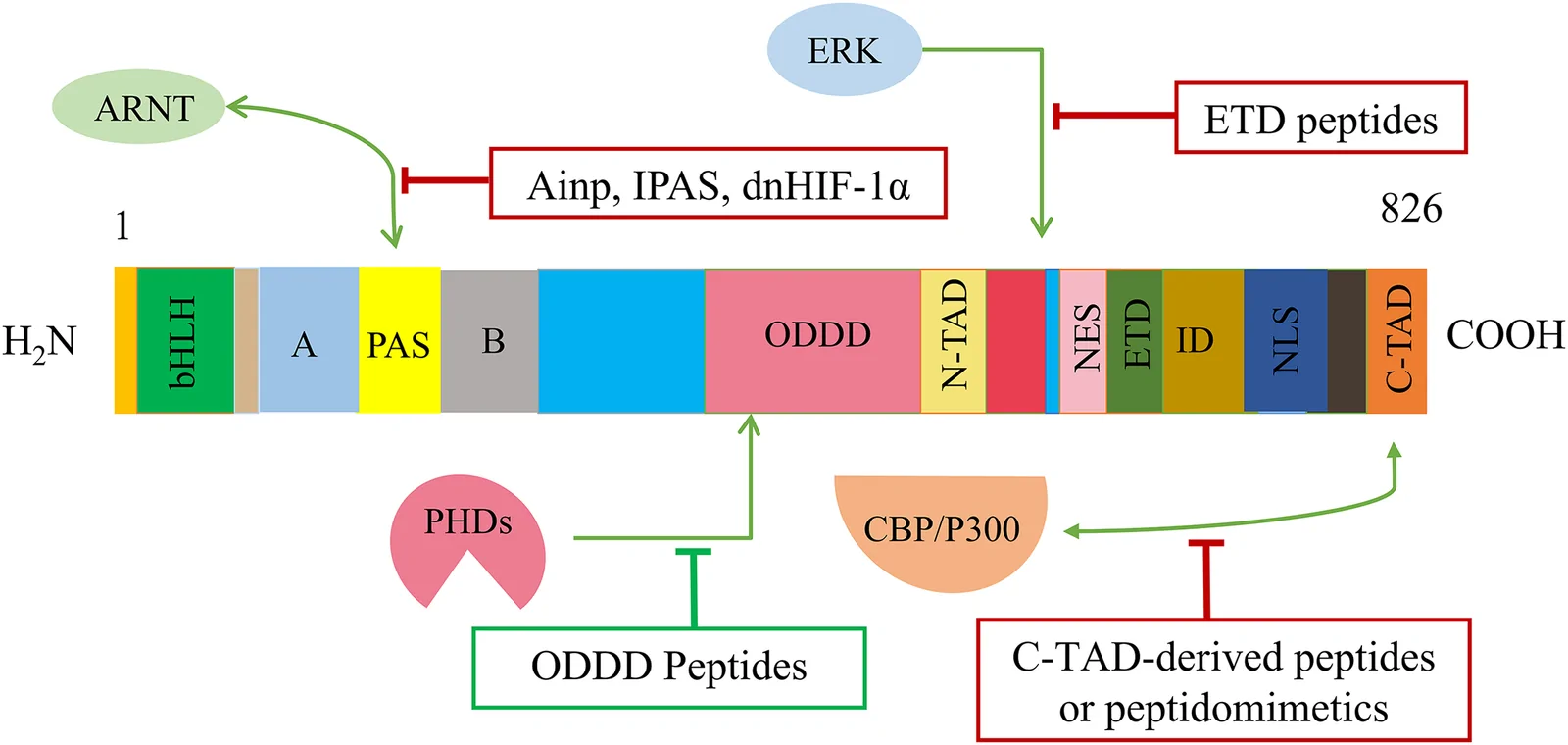
Peptide targeting of HIF-α. Peptides target the binding of certain HIF-1α domains to elements of the transcription machinery or hypoxia signaling, including PHDs, ARNT, and CBP/P300 (inhibiting peptides in red, activating peptides in green). Peptides generated from the ETD suppress the phosphorylation-dependent nuclear contacts of HIF-1α and the modification of HIF-1α by ERK. Ainp: Aryl hydrocarbon receptor-interacting protein; ARNT: Aryl hydrocarbon receptor nuclear translocator (HIF-1β subunit); bHLH: Basic Helix-Loop-Helix; CBP/P300: CREB-binding protein/E1A binding protein p300; C-TAD: C-terminal transactivation domain; dnHIF-1α: Dominant negative hypoxia-inducible factor-1α; ERK: Extracellular signal-regulated kinase; ETD: ERK targeted domain; HIF-1α: Hypoxia-inducible factor-1α; HIF-α: Hypoxia-inducible factor-α; ID: Inhibitor of DNA binding; IPAS: Inhibitory PAS domain protein (a negative regulator of HIF); NES: Nuclear export signal (sequence that mediates protein export from the nucleus); NLS: Nuclear localization signal (sequence that mediates protein import into the nucleus); N-TAD: N-terminal transactivation domains; ODDD: Oxygen-dependent degradation domain; PAS: Per-ARNT-Sim domain; PHDs: Prolyl hydroxylase domain.

Peptides modeled after HIF-α isoforms suppress or activate HIFs by competing for critical protein interactions, enabling therapeutic delivery through protein transduction using cell-permeable peptides such as HIV-1 TAT, which modify protein–protein interactions.^112^ Figure 7 shows how inhibitory (red) and activating (green) peptides target different HIF-1α domains, affecting interactions with ARNT, PHDs, and CBP/P300, thereby modulating transcriptional responses to hypoxia. HIF-1α interactors were the original targets of protein transduction technology, which was used to increase HIF activity. In *vitro* and animal studies, peptides fused to the TAT sequence generated from HIF-1α ODD enter cells, enhance HIF-1α stability, and stimulate angiogenesis.^113^ HIF-1α-originating peptide reduces HIF-1α function, affecting glucose absorption and cell proliferation in pancreatic cancer cells and animal xenograft models.^114^ In *vitro* and *in vivo,* EZN-2968, a specific HIF inhibitor that particularly affects *HIF-1α* mRNA expression, affects the regulation of *HIF-1α*-dependent genes.^115^ By preventing HIF-1α from binding to the HRE region, the anthracyclines daunorubicin and doxorubicin inhibit its transcriptional activity.^15^

Cancer treatment with monoclonal antibodies involves targeting mutant or abnormally expressed proteins, whereas PROteolysis-TArgeting Chimers (PROTACs) target non-druggable proteins using the ubiquitin-proteasome system (UPS).^116^ PROTAC-A and PROTAC-B decrease hormone-driven breast and prostate cancer development by selectively targeting androgen receptors (AR) and estrogen receptor alpha (ERα), for degradation via the pVHL E3 ubiquitin ligase system.^117^ Researchers have produced E3 ligase ligands in the form of tiny molecules, including MDM2 (mouse double minute 2 homologue), cereblon, IAPs (inhibitor of apoptosis proteins), and pVHL. Besides, numerous studies have described small molecule VHL-based PROTACs aimed at diverse proteins, including FAK, ALK, ER, SGK3, RTKs, SMARCA2/4, Smad3, cdc20, BCR-ABL, AR, and bromodomain protein (BRD) 7/9.^116^

### Indirect modulation of the hypoxia-inducible factor pathway

#### Targeting of upstream regulators

Multiple factors regulate the expression and activity of HIF-α, including microtubules, HSP90, topoisomerases, HDAC, PHDs, Trx, and PI3K/Akt/FK506 binding protein 12-rapamycin-associated protein (FRAP)/mTOR pathway downstream of growth factor receptors, which are targeted by therapeutic agents.^118^ Activation of the PI3K/Akt/FRAP/mTOR pathway, which drives cell growth and prevents apoptosis, occurs frequently in malignancies via growth factor signaling or *PTEN* gene mutations, with Wortmannin or LY294002 effectively suppressing Akt activity, thereby inhibiting HIF-1α production in mice xenografts.^119^ There are ongoing phase I clinical studies investigating MK-2206, an exciting new Akt allosteric inhibitor that has demonstrated potential in suppressing Akt phosphorylation and shrinking tumors in neuroendocrine cancer, pancreatic cancer, and melanoma.^120^ In cancer therapy, targeting HIF-α production with agents such as everolimus, KC7F2, rapamycin, and temsirolimus is promising, as they inhibit its translation promoted by the Akt/mTOR pathway, commonly upregulated by growth factors, and are either approved or currently in clinical trials.^121^ Glyceollins reduce HIF-1α translation and stability by decreasing the interaction between HSP90 and HIF-1α, as well as inhibiting the PI3K/Akt/mTOR signaling cascade.^122^ Topotecan hydrochloride, an FDA-approved camptothecin analogue, stabilizes DNA-topoisomerase I complex, suppressing HIF-1α as well as inhibiting angiogenesis through the PI3K/Akt pathway; NSC 644221 inhibits HIF-1α synthesis.^123^ In addition, multiple HDAC inhibitors such as trichostatin A, suberoylanilide hydroxamic acid, vorinostat, FK228, and NVPLAQ824 have demonstrated potential in decreasing the expression of HIF-α protein, as well as angiogenic factors like eNOS, VEGF, and VEGF receptor.^124^ Phase I clinical data revealed ENMD-1198's efficacy in maintaining prolonged disease control for ovarian, prostate, and pancreatic neuroendocrine cancers.^125^ In hypoxia, chemical PHD activators KRH102140 and KRH102053 effectively activate PHD2 and decrease mRNA and protein expressions of HIF-regulated target genes like aldolase A, VEGF, MCT4, and enolase 1, as well as HIF-1α.^126^

#### Inhibition of downstream targets

Elevated HIF-1α levels in cancer increase VEGF expression, and microvessel formation, leading to poor prognosis and chemotherapy failure. Hypoxia-regulated angiogenesis factors include NOSs, angiopoietins, fibroblast growth factors (FGFs), plasminogen activator receptors, collagen prolyl hydroxylase, and MMPs.^127^ The FDA has approved several small molecules and antibodies, including bevacizumab, sorafenib, and sunitinib, for targeting VEGF/VEGFR signaling in multiple cancer types.^128^ Bevacizumab is administered to treat multiple cancers like renal cell carcinoma, non-small cell lung cancer, glioblastoma multiforme, breast cancer, and colorectal cancer.^128^^,^^129^ In addition to presently available inhibitors and mTOR pathway inhibitors like temsirolimus, rapamycin analogues, and everolimus, immune-checkpoint blockers are the primary treatment approach for kidney cancer.^130^ PX-478 accelerates protein degradation by reducing HIF-1α transcription, translation, and de-ubiquitination.^131^ Bortezomib prevents HIF-1α transcription and translation, inhibiting the p300 recruitment.^132^ HDAC, a type of deacetylases, is classified into class I, class II, and class IV, which are dependent on Zn^2+^, and class III, which uses the co-substrate NAD^+^ to alter HIF-1α and HIF-2α stability and control HIF-dependent metabolic processes.^133^ Table 4 presents a list of additional HIF regulatory molecules and inhibitors, which expands the potential treatment targets for hypoxia-mediated malignancies beyond those addressed in this study.

**Table 4:** Several HIF regulators that were not discussed in the present study.

The control group	Compound	Target	Reference
Direct effects	Anthracyclines DXR and DNR	DNA-binding compounds that obstruct HIF-DNA interaction	179
	Polyamide	Binds to HRE	180
	Dominant negative HIF-1α vector	Disruption of HIF dimerization	181
Upstream regulators	RITA (NSC-652287)	p53-MDM2	182
Downstream of HIF target genes	5TDG 2DG 2FDG Lonidamine	Hexokinase inhibitor	183
	Glufosfamide	GLUT1 transports these molecules into cells. Cells release the active medication by cleaving the bond involving glucose and the alkylator.	184
	Sulfonamides Indisulam	CAIX and CAXII suppressor	185
	2-Glu-SNAP	Conveyed by GLUT1 inside cells	186
	CHC AR-C155858	MCT1 inhibitor	187
	Fasentin	Sensitizes cells to Fas-induced cell death by reacting with GLUT1	188

AR-C: Selective inhibitor of monocarboxylate transporters; CAIX: Carbonic anhydrase IX; CAXII: Carbonic anhydrase XII; CHC: Clathrin heavy chain; DG: Deoxyglucose; DNR: Daunorubicin; DN: Dominant negative; DXR: Doxorubicin; FDG: Fluorodeoxyglucose; GLUT: Glucose transporters; HIF-1α: Hypoxia-inducible factor-1α; HRE: Hypoxia response element; MCT: Monocarboxylate transporter; MDM2: Mouse double minute 2 homologue; NSC: National service center number; RITA: Reactivation of p53 and induction of tumor cell apoptosis; SNAP: S-nitroso-N-acetylpenicillamine; TDG: Thymine DNA glycosylase.

### Combination therapies

#### Synergy with existing treatments

Acriflavine, an HIF-1 inhibitor, increases radiosensitivity by converting endogenous H_2_O_2_ to O_2_, reducing hypoxia, and increasing ROS formation, which reducing tumor cell viability, while decreasing VEGF and MMP-9 production.^134^ SN-38 appears to enhance radiosensitivity in colorectal cancer by downregulating VEGF expression and HIF-1α, subsequently inducing cell cycle arrest at S and G2/M phases.^135^ When exposed to radiation, the inhibitor of HIF-1 vitrexin increases the vulnerability of glioma cancer stem cells (CSC) to hyperbaric oxygen.^136^

In addition, in hypoxic conditions, the synergistic cytotoxicity of sorafenib and radiation against breast CSCs, mediated through G2/M arrest, therefore preventing metastases through lowering HIF-1α and MMP-2 levels.^137^ A protease inhibitor of the human immunodeficiency virus, nelfinavir, lowered VEGF expression and HIF-1α in lung cancer, possibly enhancing radiation sensitivity.^138^ When Endostar is exposed to radiation, it decreases angiogenesis and tumor development.^139^ The natural compound chrysin demonstrates radio-sensitizing effects in triple-negative breast cancer cell lines through dual inhibition of HIF-1α and VEGF signaling pathways.^140^ Moreover, atovaquone, an anti-malarial agent, has been repurposed to inhibit mitochondrial complex III, improving radiotherapy efficacy in tumor hypoxia. The drug is undergoing trial for the treatment of patients with NSCLC (NCT0262808).^141^

**Figure 8:**
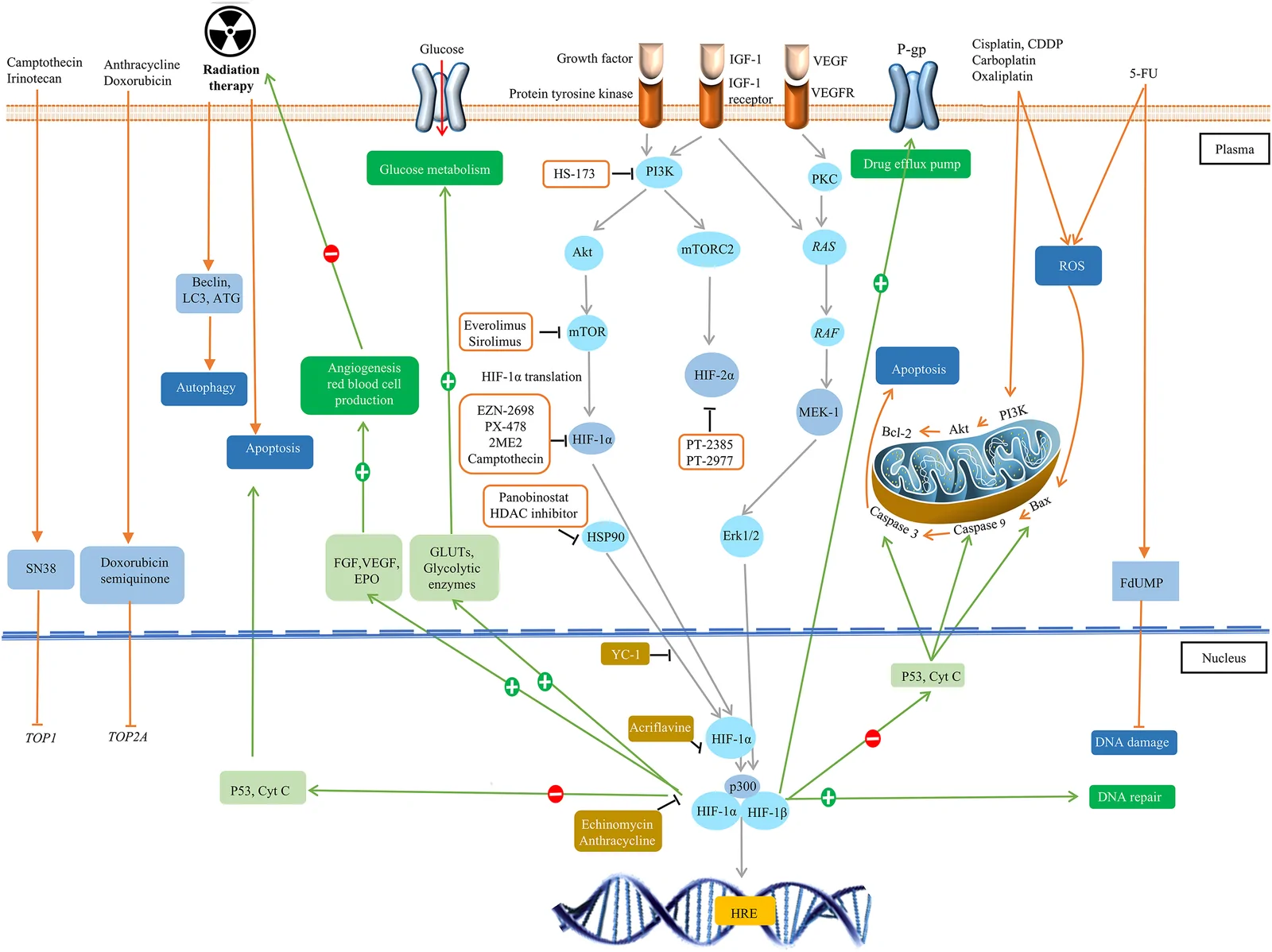
HIF-1α and HIF-2α inhibition combined with targeted treatment, radiation, and chemotherapy. Chemotherapies such as 5-FU and cisplatin can damage DNA and induce cancer cell death. Radiation therapy can cause apoptosis of cancer cells and increase ROS levels. However, the HIF-1 pathway in tumor cells is triggered by hypoxia, leading to treatment resistance. Cancer cells enhance chemo-drug efflux, perform DNA repair, block cell apoptosis, and alter cell metabolism in response to chemotherapy resistance mediated by the HIF pathway. In addition, angiogenesis and increased blood flow, which enhance cancer cell survival, produce radiation resistance. Therefore, the combination of HIF inhibitor therapy with chemotherapy, radiation, and targeted therapy has several synergistic effects by reversing the resistance via the HIF route. Akt: Protein kinase B; Bcl 2: B-cell lymphoma 2; Caspase 9: Caspase 9 (Cysteine–aspartic acid protease 9); 5-FU: 5-Fluorouracil; COOP: Cooperative protein; Cyt C: Cytochrome *c*; *EPO:* Erythropoietin; Erk 1/2: Extracellular signal-regulated kinase 1/2; FdUMP: Fluorodeoxyuridine monophosphate; FGF: Fibroblast growth factors; GLUTs: Glucose transporters; HIF-1: Hypoxia-inducible factor-1; HRE: Hypoxia response elements; HIF-1α: Hypoxia-inducible factor-1α; HSP90: Heat shock protein 90; HS 173: HS-173 (a specific PI3K inhibitor compound); *IGF1*: Insulin-like growth factor 1; LC3: Microtubule-associated protein 1A/1B-light chain 3; ATG: Autophagy-related gene; MEK 1: Mitogen-activated protein kinase 1; mTOR: Mechanistic target of rapamycin; P-gp: P-glycoprotein; mTORC2: Mechanistic target of rapamycin complex 2; P53: Tumor protein p53; PKC: Protein kinase C; *PI3K*: Phosphoinositol 3-OH kinase; PT 2977: Belzutifan (PT2977, a HIF-2α inhibitor); *Raf*: Rapidly accelerated fibrosarcoma (RAF proto-oncogene serine/threonine-protein kinase); *Ras:* Rat sarcoma viral oncogene homolog; ROS: Reactive oxygen species; SN38: 7-ethyl-10-hydroxycamptothecin (active metabolite of irinotecan); *TOP1*: DNA topoisomerase I; *TOP2A*: DNA topoisomerase II alpha; VEGF: Vascular endothelial growth factor; p300: E1A binding protein p300 (EP300); VEGFR: Vascular endothelial growth factor receptor; YC 1: YC-1 (3-(5′-hydroxymethyl-2′-furyl)-1-benzyl indazole; a HIF inhibitor compound).

Combined chemotherapy and anti-VEGF antibodies is beneficial in lung adenocarcinoma^142^ and colorectal malignancies, with *KRAS*-mutant and wild-type tumors performing equally well.^143^ The HIF-2α-specific inhibitor PT-2385, boosts the efficacy of sorafenib and decreases its side effects.^144^ As illustrated in Figure 8, dual HIF-1α/HIF-2α blockade synergizes with conventional chemotherapy, radiation, and molecularly targeted agents effectively overcoming hypoxia-induced treatment resistance by suppressing angiogenesis, affecting survival signaling pathways, and enhancing therapeutic efficacy. Moreover, combining ganetespib with immunotherapy treatment for melanoma has shown strong synergistic effects, coupled with ganetespib and anti-PD-1 (clone 29 F.1A12, 135,204). Besides, combination therapy with ganetespib and anti-CTLA-4 (9H10) enhances systemic and intratumoral CD8^+^ T-cell responses, leading to improved tumor control and survival outcomes.^145^ Prim-O-glucosyl cimifugin (POG) suppresses polymorphonuclear myeloid-derived suppressor cells (PMN-MDSC) development and metabolism, specifically targeting their immunosuppressive properties. When combined with immunotherapy, significant improvement in PD-1 inhibitor (RMP1-14) efficacy is observed, accompanied by a marked rise in tumor-infiltrating CD8^+^ T-cells in murine cancer models.^146^ Clinical trials are currently investigating combination approaches of immunotherapy with HIF inhibition in ccRCC, utilizing both non-specific HIF inhibitors like vorinostat (NCT02619253) and selective HIF-2α antagonists like PT2385 alongside anti-PD-1 therapy with nivolumab (NCT02293980).^147^ Evidence presented in Table 5, demonstrates that the addition of HIF inhibitors to conventional treatments yields improved therapeutic responses in several cancer types.

**Table 5:** Synergistic effects of combining HIF inhibitors with chemotherapy, radiation, and immunotherapy in the treatment of various cancers.

Combination therapy	Cell and cell line	Synergistic effects/mechanism	Agent associated with HIF	Reference
Chemotherapy	Brain: Glioma (D54MG)	Caspase-mediated apoptotic cell death	Knockdown of HIF-1α	139
	Head and neck: Xenografts model (SQ20B)	Reduces VEGF and alters the tumor microenvironment	Erlotinib: EGFR inhibitor	140
	Small cell lung cancer cells	Eliminates PI3K-Akt activity	NVP-ADW742: IGF1 receptor kinase inhibitor	141
	Pancreas: Cells	Induces the release of HMGB1, ATP, and CRT exposure	PX-478	189
	Colorectal: Cells	Decreases multidrug resistant protein MDR1-pg	(HT29) l-carnosine	190
	Liver: Cells (HepG2)	Induces cell apoptosis, inhibits tumor angiogenesis	2-Methoxyestradiol	148
Radiation	Colorectal cancer: HT29 and SW480	Cell cycle arrest occurs during the S and G2/M stages.	SN-38	135
	Breast cancer stem cells: MCF-7 and MDA-MB-231	Causes G2/M cell cycle arrest, prevents metastasis	Sorafenib	191
	HNSCC: FaDu and ME180 xenograft model	Enhances the need for oxygen and kills radiosensitive cells	HIF-1 knockdown	192
	Non-small cell lung cancer:	Reduces HIF-1α/VEGF expression	Nelfinavir: HIV protease inhibitor	193
	Triple-negative breast cancer: MDA-MB-231	Suppresses HIF 1α while promoting apoptosis by radiation	Chrysin	140
Immunotherapy	Melanoma: Mouse (B16F10)	Prevents HIF-1α expression	PMN-MDSC inhibitor: POG	144
	Prostate cancer: Mouse (RM-1)	Suppresses HIF-1α expression	IDF-11774	194
	Breast cancer: Mouse (4T1)			
	HCC: Human (MHCC97L PLC/PRF/5, Hep3B) mouse (Hepa1-6)	HIF-1α is upregulated when the downstream product MDSC is suppressed	*ENTPD2* inhibitor: ARL67156	153
	Colon carcinoma: Mouse (MC38/gp100)	Prevents HSP90 and causes the breakdown of HIF-1α	Ganetespib	195
	Melanoma: Human (melanoma 2338,2400, 2549,2559, 2812)			

4T1: Mouse mammary carcinoma cell line; ARL67156: A small ectonucleoside triphosphate diphosphohydrolase 2 inhibitor; ENTPD2: Ectonucleoside triphosphate diphosphohydrolase 2; ATP: Adenosine triphosphate; B16F10: Mouse melanoma cell line, subclone F10 of B16; CRT: Calreticulin; HIF 1α: Hypoxia-inducible factor 1α; D54MG: Duke 54 malignant glioma; EGFR: Epidermal growth factor receptor; Fadu: Human hypopharyngeal squamous cell carcinoma cell line; G2/M: G2/M phase in cell cycle; HCC: Hepatocellular carcinoma; Hep3B: Human epithelial protein 3B; hepg2: Human epithelioma G2 cell line; Hepa1-6: Mouse hepatoma cell line 1–6; HNSCC: Head and neck squamous cell carcinomas; HIF: Hypoxia-inducible factor; HIV: Human immunodeficiency virus; HMGB1: High mobility group box 1 protein; HSP90: Heat shock proteins 90; HT29: Colon cancer cell line; IDF 11774: Selective hypoxia-inducible factor 1α inhibitor; IGF1: Insulin-like growth factor 1; MCF 7: Michigan cancer foundation-7; MC38/gp100: Mouse colon 38/glycoprotein 100 cell line; MDA MB 231: MD Anderson-metastatic breast 231; MDR1 pg: Multidrug resistance protein 1p-glycoprotein.; ME180: Human cervical cancer cell line; NVP ADW742: Novartis compound ADW742, a selective insulin-like growth factor 1 receptor kinase inhibitor; PI3K Akt: Phosphoinositol 3-OH kinase/protein kinase B; PMN MDSC: Polymorphonuclear myeloid-derived suppressor cells; POG: Polymorphonuclear myeloid-derived suppressor cells inhibitor; PX 478: Small molecule inhibitor of hypoxia-inducible factor 1α; RM 1: Mouse prostate cancer cell line derived from Ras + Myc-induced tumor; S: Synthesis phase in cell cycle; SN38: Active metabolite of irinotecan, topoisomerase I inhibitor; SQ20B: Subcutaneous tumor model in mice using SQ20B cells; SW480: Colon cancer cell line; VEGF: Vascular endothelial growth factor.

#### Overcoming resistance

Research suggests that radiation-upregulated Notch pathway enhancing radioresistance in lung cancer via HIF-1α activation. The HIF inhibitor YC-1 suppresses this upregulation, demonstrating therapeutic potential.^148^ Recent findings reveal that placental growth factor (PIGF's) essential role in maintaining protective vascular networks through p53-regulated paracrine signaling, which mediates ionizing radiation (IR) resistance. This resistance mechanism establishes PlGF as a promising therapeutic target for radio-sensitization strategies in combination with radiation therapy. In addition, tumor cell signaling-directed drugs may help regulate tumor vasculature, reducing radiation resistance.^149^ Patients with HNSCC who receive chemotherapy and radiation therapy have higher levels of inhibitory immunological Treg cells, which causes them to become radioresistant.^150^ PD-1 controls T cell responses and inhibits autoimmunity. When combined with radiation therapy, anti-programmed death-ligand 1 (PD-L1) antibodies provide abscopal impacts and primary tumor regression, with high response rates and few adverse effects.^151^ The interconnected regulation of PD-L1 by HIF-1α under hypoxia reveals a potential adaptive response that maintains immunosuppressive tumor microenvironments despite radiation exposure.^152^ Combined inhibition of HIF-1α and PD-L1 signaling pathways mitigates radioresistance while reversing immunosuppression, achieved via vascular normalization that increases blood flow and alleviates tumor hypoxia.^153^ In phase 3 trials, the anti-VEGF antibody bevacizumab demonstrated tumor-type dependent efficacy, with breast cancer patients (6.7% response) showing greater sensitivity than colorectal cancer patients (3.3% response).^154^ Therefore, combining these techniques with radio-chemotherapy and hypoxia-targeted drugs allows precise targeting of the most hypoxic and treatment-resistant tumor regions, significantly improving therapeutic outcomes.

## Clinical implications and biomarkers

The HIF pathway plays a key role in tumor adaptation to low-oxygen (hypoxic) environments and represents an important predictive biomarker of the effectiveness of anti-angiogenic therapies. HIFs control gene expressions involving genes that are central to processes such as new blood vessel formation, cellular metabolism, and survival. Of special mention, HIF-1α is frequently found at higher levels in various types of tumors.^155^ Moreover, genetic mutations in human neoplasms especially the inactivation of the *VHL* gene in ccRCC, can induce the activation of HIFs.^11^ Hypoxia-related gene signature, including *VEGFA,* GLUT1(*SLC2A1*), and *LDHA* have been reported to be associated with poor clinical outcomes and drug resistance in various cancers, with a high hypoxia gene signature causing decreased survival and limited response to anti-VEGF therapies.^156^ Targeting the HIF pathway, alongside anti-angiogenic therapies, has shown promise in preclinical models. Folkman et al.^157^ demonstrated that inhibiting HIF-1α could restore sensitivity to anti-VEGF therapies, suggesting that combination strategies may be beneficial. Further, VEGF and carbonic anhydrase 9, two HIF-regulated factors, have been identified as promising biomarkers in liquid biopsies for monitoring tumor hypoxia and assessing therapy response, with elevated levels related to angiogenesis and tumor progression.^158^ Measurement of these biomarkers allows clinicians to modulate therapeutic regimens in accordance with the degree of oxygen shortage in the tumor, therefore optimizing patient care efficacy.

The conventional method of quantifying tumor hypoxia is to evaluate the partial pressure of oxygen in tissue. This invasive method involves polarographic histography of pO2 through direct implantation of an Eppendorf electrode into the tissue of interest. Höckel and Vaupel performed the initial testing in the early 1990s.^158^ Disrupting STAT3-mediated autophagy represents a novel therapeutic approach because this pathway is important for cancer cell adaptation and resistance, and inhibiting it may improve outcomes in patients with hypoxia-driven cancers.^159^ Non-invasive detection of hypoxia in tissues and the TME may prove useful in treatment enhancement and determining which cases are resistant or unresponsive to a specific medication. At present, there are several clinically available imaging modalities and those being investigated.^160^ A number of tracers, with specific strengths and particular uses in positron emission tomography (PET) imaging, have been established to identify hypoxic tumor areas. Development of the 2-nitroimidazoles as radiolabeled probes for hypoxia detection was based on findings that 14C-labeled N-alkyl-2-nitroimidazole molecules selectively bind to cells under low oxygen environments.^161^ Additional evidence suggests that oxygenation-enhanced MRI or so-called blood oxygenation-dependent or tumor oxygenation level-dependent MRI could be a superior option for imaging hypoxia. Compared to PET scanning, MRI approaches offer a cost-effective and easily accessible method for revealing hypoxic regions in tumors without using radiolabeled tracers.^162^ An alternative MRI technique investigated globally for detecting localized tumor hypoxia is MRI-Fluorine.^163^ In addition, hypoxia can be visualized using electron paramagnetic resonance imaging (EPRI), also referred to electron spin resonance (ESR). EPRI/ESR measures unpaired electron spins of freely diffusible oxygen molecules using an injected spin probe to directly assess relaxation times, as opposed to MRI, which images spatial proton distribution.^164^

## Future directions and emerging research

Although significant progress has been made in characterizing HIF signaling and its function in cancer, numerous challenges remain with much to be explored in the future. One key goal is creating highly selective inhibitors targeting a single HIF isoform, either HIF-1α or HIF-2α, to reduce off-target effects and enhance efficacy. Because resistance to HIF-targeted therapies may arise through compensatory pathways or tumor heterogeneity, future research should focus on unraveling the molecular processes that cause resistance and discovering predictive biomarkers that guide patient selection and treatment response. Combination of a number of omics strategies—genomics, proteomics, transcriptomics, and metabolomics—will be the major determinant in obtaining an integrated perspective of HIF signaling pathways and their specific functions in different cancers. In addition, these approaches can help unravel new therapeutic targets and guide the logical design of combination therapies. Particularly, combining HIF inhibitors with conventional treatments like chemotherapy, radiation, immunotherapy, or anti-angiogenic drugs shows great potential for overcoming resistance and improving clinical results.

Emerging technologies like single-cell sequencing, spatial transcriptomics, and high-performance imaging are enabling scientists to examine the tumor microenvironment (TME) in greater detail than ever before. These technologies reveal insights into the impact of hypoxia and hypoxia-inducible factors (HIFs) on cell-to-cell communication, immune escape, and metastatic potential. Artificial intelligence and machine learning approaches also enable greater ability for the prediction of patient response and the planning of tailored treatment plans. Beyond the field of cancer research, interest in the role of HIFs in many non-malignant conditions such as ischemic damage, inflammatory conditions, and metabolic disease is also increasing, possibly laying the ground for novel therapeutic strategies. Future clinical trials must emphasize the incorporation of rigorous correlative research, longitudinal biomarker evaluations, and patient classification according to hypoxic signatures to expedite the translation of laboratory findings into successful, personalized cancer treatments. Broadening our knowledge of HIF biology using multidisciplinary approaches and state-of-the-art technologies is essential to the development of the next generation of therapies. These advances hold out the potential of reducing cancer patient mortality and enhancing quality of life for cancer patients, as well as offering potential therapeutic gain for the treatment of other hypoxia-associated diseases.

## Study limitations

Although this review extensively discusses the significance of HIF signaling in cancer and therapeutic resistance, various limitations must be addressed. Firstly, developing universal therapeutic approaches is challenging because HIF-1α and HIF-2α exhibit dual functions across various cancer types. Secondly, HIF-targeted therapies face a major challenge due to the inconsistency between preclinical results and clinical efficacy. Furthermore, the review recognizes efflux pump activation and other HIF-mediated drug resistance pathways but excludes resistance specific to HIF inhibitors, which is a critical clinical development gap. To develop targeted cancer therapeutics, these challenges highlight the necessity of future research that integrates multi-omics methodologies, clinical testing, and systems-level assessment of HIF signaling networks.

## Conclusion

HIFs play crucial roles in cancer biology, angiogenesis, regulating tumor development, metastasis, invasion, metabolic reprogramming, and therapeutic resistance. The hypoxic tumor microenvironment upregulates HIF-1α and HIF-2α, which regulate genes associated with metabolic adaptation, cell proliferation, and angiogenesis. Their complicated signaling networks, particularly HIF-1α and HIF-2α, present promising therapeutic targets in cancer treatment. Furthermore, HIF-1α increases the important genes expression, such as *VEGFA, IGF2*, and *TGFB1*, a crucial role in angiogenesis. HIF promotes the Warburg effect by redirecting cellular metabolism toward glycolysis, even when oxygen is readily available. Understanding HIF signaling molecular pathways can assist in creating tailored drugs and enhancing patient outcomes. Some approved drugs indirectly impact the HIF-1/2α pathway, demonstrating the extensive crosstalk linking HIF signaling with other biological pathways. With increasing understanding of HIF structures and interactions, the development of more effective inhibitors of HIF-1/2 may become possible in the near future.

Existing research indicates that HIF targeting may be a promising strategy for cancer treatment. Diverse approaches to suppress HIF activity or interfere with its downstream signaling pathways are under investigation. These may result in combination therapies with improved therapeutic efficacy. Modalities such as small molecule inhibitors, peptidomimetics, antibodies, targeting of upstream or downstream regulators, and hypoxia-activated prodrugs have been developed to limit HIF function, which leads to changes in how cancer cells adapt to hypoxic conditions. Preclinical investigations and early-stage clinical investigations have yielded encouraging outcomes, indicating the potential of HIF inhibitors in diminishing tumor growth and enhancing patient outcomes. Upcoming research must concentrate on identifying the precise roles of different HIF isoforms across diverse cancer types to further tailor treatment strategies.

Preclinical investigations suggest that the efficacy of conventional therapies, including chemotherapy and radiotherapy, can be increased through HIF inhibition, possibly overcoming resistance. The involvement of HIF signaling in the TME offers insights into patient prognosis and therapy responses, facilitating tailored treatment regimens. Current research aims to elucidate the molecular mechanisms of HIF signaling, identify biomarkers for predictive response, develop selective HIF-1/2α inhibitors, and explore drug combinations that yield additive or synergistic effects for sustained efficacy in chemotherapeutic agents. By overcoming existing difficulties and utilizing developments such as the development of selective HIF inhibitors, targeted therapies, immunotherapy, and precision medicine, we aim to achieve improved outcomes for patients with cancer and progress towards a cure for this deadly disease.

## Authors contribution

Abdul Halim Shaikat: conceptualization, writing – original draft, data extraction, drawing, and data analysis; S.M. Asadul Karim Azad: writing – original draft, drawing figures, and data extraction; Md. Azizur Rahman Tamim: writing – original draft, figure drawing; Mohammed Sailim Ullah: writing – original draft; Mofazzal K. Sabbir: writing – original draft; Md Towhidul Islam Tarun: writing – original draft; Shohana Sabrin: writing – original draft; Md Zihad Mahmud: writing – original draft; Mohammad Nurul Amin: manuscript revision; Md Ashiq Mahmud: conceptualization, supervision, and manuscript revision. All authors reviewed and approved the submission of the final version of the manuscript.

## Ethics statement

None.

## Data availability statement

The datasets used in this study can be obtained from the corresponding author upon reasonable request.

## Declaration of generative AI and AI-assisted technologies in the writing process

The authors declare that generative artificial intelligence (AI) and AI assisted technologies were not used in the writing process or any other process during the preparation of this manuscript.

## Funding

None.

## Conflict of interest

The authors declare that they have no known competing financial interests or personal relationships that could have appeared to influence the work reported in this paper.

## References

[1] Li Y., Zhao L., Li X.F. (2021). Hypoxia and the tumor microenvironment. Technol Cancer Res Treat.

[2] Kane A.D., Kothmann E., Giussani D.A. (2020). Detection and response to acute systemic hypoxia. BJA Educ.

[3] Rey S., Schito L., Koritzinsky M., Wouters B.G. (2017). Molecular targeting of hypoxia in radiotherapy. Adv Drug Deliv Rev.

[4] Challapalli A., Carroll L., Aboagye E.O. (2017). Molecular mechanisms of hypoxia in cancer. Clin Transl Imaging.

[5] Vaupel P., Harrison L. (2004). Tumor hypoxia: Causative factors, compensatory mechanisms, and cellular response. Oncologist.

[6] Emami Nejad A., Najafgholian S., Rostami A. (2021). The role of hypoxia in the tumor microenvironment and development of cancer stem cell: A novel approach to developing treatment. Cancer Cell Int.

[7] Lloyd M.C., Cunningham J.J., Bui M.M., Gillies R.J., Brown J.S., Gatenby R.A. (2016). Darwinian dynamics of intratumoral heterogeneity: Not solely random mutations but also variable environmental selection forces. Cancer Res.

[8] Semenza G.L. (2023). Regulation of erythropoiesis by the hypoxia-inducible factor pathway: Effects of genetic and pharmacological perturbations. Annu Rev Med.

[9] Kim S.-H., Oh G.-S., Sohn W.-M., Lee K., Yang H.-J., Bae Y.-A. (2018). Molecular characteristics and induction profiles of hypoxia-inducible factor-1α and other basic helix–loop–helix and Per–Arnt–Sim domain-containing proteins identified in a carcinogenic liver fluke Clonorchis sinensis. Parasitology.

[10] Semenza G.L. (2014). Oxygen sensing, hypoxia-inducible factors, and disease pathophysiology. Annu Rev Pathol.

[11] Majmundar A.J., Wong W.J., Simon M.C. (2010). Hypoxia-inducible factors and the response to hypoxic stress. Mol Cell.

[12] Brahimi-Horn M.C., Chiche J., Pouysségur J. (2007). Hypoxia and cancer. J Mol Med (Berl).

[13] Semenza G.L. (2012). Hypoxia-inducible factors: Mediators of cancer progression and targets for cancer therapy. Trends Pharmacol Sci.

[14] Lee K., Zhang H., Qian D.Z., Rey S., Liu J.O., Semenza G.L. (2009). Acriflavine inhibits HIF-1 dimerization, tumor growth, and vascularization. Proc Natl Acad Sci U S A.

[15] Lee K., Qian D.Z., Rey S., Wei H., Liu J.O., Semenza G.L. (2009). Anthracycline chemotherapy inhibits HIF-1 transcriptional activity and tumor-induced mobilization of circulating angiogenic cells. Proc Natl Acad Sci U S A.

[16] Lau C.K., Yang Z.F., Ho D.W. (2009). An Akt/hypoxia-inducible factor-1α/platelet-derived growth factor-BB autocrine loop mediates hypoxia-induced chemoresistance in liver cancer cells and tumorigenic hepatic progenitor cells. Clin Cancer Res.

[17] Wu M.-Z., Tsai Y.-P., Yang M.-H. (2011). Interplay between HDAC3 and WDR5 is essential for hypoxia-induced epithelial-mesenchymal transition. Mol Cell.

[18] Lee J.W., Yang D.H., Park S. (2018). Trichostatin a resistance is facilitated by HIF-1α acetylation in HeLa human cervical cancer cells under normoxic conditions. Oncotarget.

[19] Rashid M., Zadeh L.R., Baradaran B. (2021). Up-down regulation of HIF-1α in cancer progression. Gene.

[20] Ali M.L., Roky A.H., Azad S.M.A.K. (2024). Autophagy as a targeted therapeutic approach for skin cancer: Evaluating natural and synthetic molecular interventions. Cancer Pathog Ther.

[21] Ajduković J. (2016). HIF-1—a big chapter in the cancer tale. Exp Oncol.

[22] Sarkar S., Sinha S., Saluja R., Kalra N. (2023).

[23] Wiesener M.S., Jürgensen J.S., Rosenberger C. (2003). Widespread, hypoxia-inducible expression of HIF-2α in distinct cell populations of different organs. FASEB J.

[24] Makino Y., Cao R., Svensson K. (2001). Inhibitory PAS domain protein is a negative regulator of hypoxia-inducible gene expression. Nature.

[25] Krishnan A., Ansari H.K. (2023). Molecular basis of response to hypoxia. Springer Nature Singapore.

[26] Depping R., Steinhoff A., Schindler S.G. (2008). Nuclear translocation of hypoxia-inducible factors (HIFs): Involvement of the classical importin α/β pathway. Biochim Biophys Acta.

[27] Hu C.-J., Sataur A., Wang L., Chen H., Simon M.C. (2007). The N-terminal transactivation domain confers target gene specificity of hypoxia-inducible factors HIF-1α and HIF-2α. Mol Biol Cell.

[28] Wang V., Davis D.A., Haque M., Huang L.E., Yarchoan R. (2005). Differential gene up-regulation by hypoxia-inducible factor-1α and hypoxia-inducible factor-2α in HEK293T cells. Cancer Res.

[29] Taylor S.E., Bagnall J., Mason D., Levy R., Fernig D.G., See V. (2016). Differential sub-nuclear distribution of hypoxia-inducible factors (HIF)-1 and -2 alpha impacts on their stability and mobility. Open Biol.

[30] Yang G., Shi R., Zhang Q. (2020). Hypoxia and oxygen-sensing signaling in gene regulation and cancer Progression. Int J Mol Sci.

[31] Fletcher S.C., Coleman M.L. (2020). Human 2-oxoglutarate-dependent oxygenases: Nutrient sensors, stress responders, and disease mediators. Biochem Soc Trans.

[32] Ortmann B.M. (2024). Hypoxia-inducible factor in cancer: from pathway regulation to therapeutic opportunity. BMJ Oncol.

[33] Bruick R.K., McKnight S.L. (2001). A conserved family of prolyl-4-hydroxylases that modify HIF. Science.

[34] Lando D., Peet D.J., Whelan D.A., Gorman J.J., Whitelaw M.L. (2002). Asparagine hydroxylation of the HIF transactivation domain a hypoxic switch. Science.

[35] Hewitson K.S., McNeill L.A., Riordan M.V. (2002). Hypoxia-inducible factor (HIF) asparagine hydroxylase is identical to factor inhibiting HIF (FIH) and is related to the cupin structural family. J Biol Chem.

[36] Sang N., Jie F., Vickram S., Irene L., Caro J. (2002). Carboxyl-terminal transactivation activity of hypoxia-inducible factor 1α is governed by a von Hippel-Lindau protein-independent, hydroxylation-regulated association with p300/CBP. Mol Cell Biol.

[37] Sif S. (2004). ATP-dependent nucleosome remodeling complexes: Enzymes tailored to deal with chromatin. J Cell Biochem.

[38] Koivunen P., Hirsilä M., Günzler V., Kivirikko K.I., Myllyharju J. (2004). Catalytic properties of the asparaginyl hydroxylase (FIH) in the oxygen sensing pathway are distinct from those of its prolyl 4-hydroxylases. J Biol Chem.

[39] Schofield C.J., Ratcliffe P.J. (2004). Oxygen sensing by HIF hydroxylases. Nat Rev Mol Cell Biol.

[40] Conway E.M., Collen D., Carmeliet P. (2001). Molecular mechanisms of blood vessel growth. Cardiovasc Res.

[41] Abhinand C.S., Raju R., Soumya S.J., Arya P.S., Sudhakaran P.R. (2016). VEGF-A/VEGFR2 signaling network in endothelial cells relevant to angiogenesis. J Cell Commun Signal.

[42] Soni S., Padwad Y.S. (2017). HIF-1 in cancer therapy: Two decade long story of a transcription factor. Acta Oncol.

[43] Semenza G.L. (2003). Targeting HIF-1 for cancer therapy. Nat Rev Cancer.

[44] Zhang Z., Yao L., Yang J., Wang Z., Du G. (2018). PI3K/Akt and HIF-1 signaling pathway in hypoxia-ischemia. Mol Med Rep.

[45] Kierans S.J., Taylor C.T. (2021). Regulation of glycolysis by the hypoxia-inducible factor (HIF): Implications for cellular physiology. J Physiol.

[46] Wenger R.H. (2002). Cellular adaptation to hypoxia: O2-sensing protein hydroxylases, hypoxia-inducible transcription factors, and O2-regulated gene expression. FASEB J.

[47] Park H.S., Kim J.H., Sun B.K., Song S.U., Suh W., Sung J.H. (2016). Hypoxia induces glucose uptake and metabolism of adipose-derived stem cells. Mol Med Rep.

[48] Yang L., Liu Q., Lu Q. (2024). Scavenger receptor class B type I deficiency induces iron overload and ferroptosis in renal tubular epithelial cells via hypoxia-inducible factor-1α/transferrin receptor 1 signaling pathway. Antioxidants Redox Signal.

[49] Kietzmann T., Mennerich D., Dimova E.Y. (2016). Hypoxia-inducible factors (HIFs) and phosphorylation: Impact on stability, localization, and transactivity. Front Cell Dev Biol.

[50] Xu Q., Briggs J., Park S. (2005). Targeting Stat3 blocks both HIF-1 and VEGF expression induced by multiple oncogenic growth signaling pathways. Oncogene.

[51] Liu L., Bai J., Hu L., Jiang D. (2023). Hypoxia-mediated activation of hypoxia-inducible factor-1α in triple-negative breast cancer: A review. Medicine.

[52] Rankin E.B., Giaccia A.J. (2016). Hypoxic control of metastasis. Science.

[53] Koshikawa N., Hayashi J.-I., Nakagawara A., Takenaga K. (2009). Reactive oxygen species-generating mitochondrial DNA mutation up-regulates hypoxia-inducible factor-1α gene transcription via phosphatidylinositol 3-kinase-Akt/protein kinase C/histone deacetylase pathway. J Biol Chem.

[54] Iommarini L., Porcelli A.M., Gasparre G., Kurelac I. (2017). Non-canonical mechanisms regulating hypoxia-inducible factor 1 alpha in cancer. Front Oncol.

[55] Li M., Wei X., Xiong J., Feng J.W., Zhang C.S., Lin S.C. (2023). Hierarchical inhibition of mTORC1 by glucose starvation-triggered AXIN lysosomal translocation and by AMPK. Life Metab.

[56] Manuelli V., Pecorari C., Filomeni G., Zito E. (2022). Regulation of redox signaling in HIF-1-dependent tumor angiogenesis. FEBS J.

[57] Dor Y., Porat R., Keshet E. (2001). Vascular endothelial growth factor and vascular adjustments to perturbations in oxygen homeostasis. Am J Physiol Cell Physiol.

[58] Bui B.P., Nguyen P.L., Lee K., Cho J. (2022). Hypoxia-inducible factor-1: A novel therapeutic target for the management of cancer, drug resistance, and cancer-related pain. Cancers.

[59] Cunningham F., Tine V.B., Paul C., Imre L., FJH M., Stitt A.W. (2019). The placental growth factor pathway and its potential role in macular degenerative disease. Curr Eye Res.

[60] Liu Q., Xu Z., Mao S. (2015). Effect of hypoxia on hypoxia inducible factor-1α, insulin-like growth factor I and vascular endothelial growth factor expression in hepatocellular carcinoma HepG2 cells. Oncol Lett.

[61] Dudley A.C., Griffioen A.W. (2023). Pathological angiogenesis: Mechanisms and therapeutic strategies. Angiogenesis.

[62] Liu Z.-L., Chen H.-H., Zheng L.-L., Sun L.-P., Shi L. (2023). Angiogenic signaling pathways and anti-angiogenic therapy for cancer. Signal Transduct Targeted Ther.

[63] Hicklin D.J., Ellis L.M. (2005). Role of the vascular endothelial growth factor pathway in tumor growth and angiogenesis. J Clin Oncol.

[64] Schito L. (2019). Hypoxia-dependent angiogenesis and lymphangiogenesis in cancer. Adv Exp Med Biol.

[65] Li S., Wei M., Zhou Z., Wang B., Zhao X., Zhang J. (2012). SDF-1α induces angiogenesis after traumatic brain injury. Brain Res.

[66] Vander Heiden M.G., Cantley L.C., Thompson C.B. (2009). Understanding the Warburg effect: The metabolic requirements of cell proliferation. Science.

[67] Semenza G.L. (2010). HIF-1: Upstream and downstream of cancer metabolism. Curr Opin Genet Dev.

[68] Chen J.-Q., Russo J. (2012). Dysregulation of glucose transport, glycolysis, TCA cycle and glutaminolysis by oncogenes and tumor suppressors in cancer cells. Biochim Biophys Acta.

[69] Nagy M. (2011). HIF-1 is the commander of gateways to cancer. J Cancer Sci Ther.

[70] Urbańska K., Orzechowski A. (2019). Unappreciated role of LDHA and LDHB to control apoptosis and autophagy in tumor cells. Int J Mol Sci.

[71] Pérez de Heredia F., Wood I.S., Trayhurn P. (2010). Hypoxia stimulates lactate release and modulates monocarboxylate transporter (MCT1, MCT2, and MCT4) expression in human adipocytes. Pflügers Archiv.

[72] MacDonald V.E., Howe L.J. (2009). Histone acetylation: Where to go and how to get there. Epigenetics.

[73] Papandreou I., Cairns R.A., Fontana L., Lim A.L., Denko N.C. (2006). HIF-1 mediates adaptation to hypoxia by actively downregulating mitochondrial oxygen consumption. Cell Metab.

[74] Fukuda R., Zhang H., Kim J-w, Shimoda L., Dang C.V., Semenza Gregg L. (2007). HIF-1 regulates cytochrome oxidase subunits to optimize efficiency of respiration in hypoxic cells. Cell.

[75] Zhang H., Gao P., Fukuda R. (2007). HIF-1 inhibits mitochondrial biogenesis and cellular respiration in VHL-deficient renal cell carcinoma by repression of C-MYC activity. Cancer Cell.

[76] Frezza C., Zheng L., Tennant D.A. (2011). Metabolic profiling of hypoxic cells revealed a catabolic signature required for cell survival. PLoS One.

[77] Chae Y.C., Vaira V., Caino M.C. (2016). Mitochondrial Akt regulation of hypoxic tumor reprogramming. Cancer Cell.

[78] Roberts David J., Tan-Sah Valerie P., Ding Eric Y., Smith Jeffery M., Miyamoto S. (2014). Hexokinase-II positively regulates glucose starvation-induced autophagy through TORC1 inhibition. Mol Cell.

[79] Lee J.M., Dedhar S., Kalluri R., Thompson E.W. (2006). The epithelial–mesenchymal transition: New insights in signaling, development, and disease. J Cell Biol.

[80] Graff J.R., Gabrielson E., Fujii H., Baylin S.B., Herman J.G. (2000). Methylation patterns of the E-cadherin 5′ CpG island are unstable and reflect the dynamic, heterogeneous loss of E-cadherin expression during metastatic progression. J Biol Chem.

[81] Whiteman E.L., Liu C.J., Fearon E.R., Margolis B. (2008). The transcription factor snail represses Crumbs 3 expression and disrupts apico-basal polarity complexes. Oncogene.

[82] Sun S., Ning X., Zhang Y. (2009). Hypoxia-inducible factor-1α induces Twist expression in tubular epithelial cells subjected to hypoxia, leading to epithelial-to-mesenchymal transition. Kidney Int.

[83] Zhang J., Cheng Q., Zhou Y., Wang Y., Chen X. (2013). Slug is a key mediator of hypoxia induced cadherin switch in HNSCC: Correlations with poor prognosis. Oral Oncol.

[84] Natarajan S., Foreman K.M., Soriano M.I. (2019). Collagen remodeling in the hypoxic tumor-mesothelial niche promotes ovarian cancer metastasis. Cancer Res.

[85] Yang J.-S., Chiao-Wen L., Shih-Chi S., Yang S.-F. (2016). Pharmacodynamic considerations in the use of matrix metalloproteinase inhibitors in cancer treatment. Expert Opin Drug Metab Toxicol.

[86] Roy S., Kumaravel S., Sharma A., Duran C.L., Bayless K.J., Chakraborty S. (2020). Hypoxic tumor microenvironment: Implications for cancer therapy. Exp Biol Med (Maywood).

[87] Ozel I., Duerig I., Domnich M., Lang S., Pylaeva E., Jablonska J. (2022). The good, the bad, and the ugly: Neutrophils, angiogenesis, and cancer. Cancers.

[88] Sáenz-de-Santa-María I., Celada L., San José Martínez A., Cubiella T., Chiara M.-D. (2020). Blockage of squamous cancer cell collective invasion by FAK inhibition is released by CAFs and MMP-2. Cancers.

[89] Vaupel P. (2004). The role of hypoxia-induced factors in tumor progression. Oncologist.

[90] Kaplan R.N., Riba R.D., Zacharoulis S. (2005). VEGFR1-positive haematopoietic bone marrow progenitors initiate the pre-metastatic niche. Nature.

[91] Erler J.T., Bennewith K.L., Cox T.R. (2009). Hypoxia-induced lysyl oxidase is a critical mediator of bone marrow cell recruitment to form the premetastatic niche. Cancer Cell.

[92] Duda D.G., Jain R.K. (2010). Premetastatic lung “niche”: Is vascular endothelial growth factor receptor 1 activation required?. Cancer Res.

[93] Nozawa H., Chiu C., Hanahan D. (2006). Infiltrating neutrophils mediate the initial angiogenic switch in a mouse model of multistage carcinogenesis. Proc Natl Acad Sci U S A.

[94] Schelter F., Halbgewachs B., Bäumler P. (2011). Tissue inhibitor of metalloproteinases-1-induced scattered liver metastasis is mediated by hypoxia-inducible factor-1α. Clin Exp Metastasis.

[95] Kim M., Park S.C., Lee D.Y. (2021). Glycyrrhizin as a nitric oxide regulator in cancer chemotherapy. Cancers (Basel).

[96] Yu G., Chen X., Chen S., Ye W., Hou K., Liang M. (2015). Arsenic trioxide reduces chemo-resistance to 5-fluorouracil and cisplatin in HBx-HepG2 cells via complex mechanisms. Cancer Cell Int.

[97] Mao Q., Unadkat J.D. (2015). Role of the breast cancer resistance protein (BCRP/ABCG2) in drug transport—an update. AAPS J.

[98] Bolderson E., Richard D.J., Zhou B.-B.S., Khanna K.K. (2009). Recent advances in cancer therapy targeting proteins involved in DNA double-strand break repair. Clin Cancer Res.

[99] Bridges E.M., Harris A.L. (2011). The angiogenic process as a therapeutic target in cancer. Biochem Pharmacol.

[100] Loges S., Schmidt T., Carmeliet P. (2010). Mechanisms of resistance to anti-angiogenic therapy and development of third-generation anti-angiogenic drug candidates. Genes Cancer.

[101] Li J.-L., Sainson R.C.A., Oon C.E. (2011). DLL4-Notch signaling mediates tumor resistance to anti-VEGF therapy *in vivo*. Cancer Res.

[102] Welti J., Loges S., Dimmeler S., Carmeliet P. (2013). Recent molecular discoveries in angiogenesis and antiangiogenic therapies in cancer. J Clin Investig.

[103] Daijo H., Kai S., Tanaka T. (2011). Fentanyl activates hypoxia-inducible factor 1 in neuronal SH-SY5Y cells and mice under non-hypoxic conditions in a μ-opioid receptor-dependent manner. Eur J Pharmacol.

[104] Xuan Y., Hur H., Ham I.-H. (2014). Dichloroacetate attenuates hypoxia-induced resistance to 5-fluorouracil in gastric cancer through the regulation of glucose metabolism. Exp Cell Res.

[105] Fallah J., Rini B.I. (2019). HIF inhibitors: Status of current clinical development. Curr Oncol Rep.

[106] Helbig L., Koi L., Brüchner K. (2014). BAY 87–2243, a novel inhibitor of hypoxia-induced gene activation, improves local tumor control after fractionated irradiation in a schedule-dependent manner in head and neck human xenografts. Radiat Oncol.

[107] Scheuermann T.H., Li Q., Ma H.-W. (2013). Allosteric inhibition of hypoxia inducible factor-2 with small molecules. Nat Chem Biol.

[108] Jones D.T., Harris A.L. (2006). Identification of novel small-molecule inhibitors of hypoxia-inducible factor-1 transactivation and DNA binding. Mol Cancer Therapeut.

[109] Scheuermann T.H., Tomchick D.R., Machius M., Guo Y., Bruick R.K., Gardner K.H. (2009). Artificial ligand binding within the HIF2α PAS-B domain of the HIF2 transcription factor. Proc Natl Acad Sci U S A.

[110] Lee K., Kim H.M. (2011). A novel approach to cancer therapy using PX-478 as a HIF-1α inhibitor. Arch Pharm Res (Seoul).

[111] Li X., Hattori A., Takahashi S., Goto Y., Harada H., Kakeya H. (2020). Ubiquitin carboxyl-terminal hydrolase L1 promotes hypoxia-inducible factor 1-dependent tumor cell malignancy in spheroid models. Cancer Sci.

[112] Rizzuti M., Nizzardo M., Zanetta C., Ramirez A., Corti S. (2015). Therapeutic applications of the cell-penetrating HIV-1 Tat peptide. Drug Discov Today.

[113] Mylonis I., Chachami G., Simos G. (2021). Specific inhibition of HIF activity: Can peptides lead the way?. Cancers.

[114] Chen J., Zhao S., Nakada K. (2003). Dominant-negative hypoxia-inducible factor-1α reduces tumorigenicity of pancreatic cancer cells through the suppression of glucose metabolism. Am J Pathol.

[115] Greenberger L.M., Horak I.D., Filpula D. (2008). A RNA antagonist of hypoxia-inducible factor-1α, EZN-2968, inhibits tumor cell growth. Mol Cancer Therapeut.

[116] Liu J., Ma J., Liu Y. (2020). PROTACs: A novel strategy for cancer therapy. Semin Cancer Biol.

[117] Rodriguez-Gonzalez A., Cyrus K., Salcius M. (2008). Targeting steroid hormone receptors for ubiquitination and degradation in breast and prostate cancer. Oncogene.

[118] Jones D.T., Harris A.L. (2012). Small-molecule inhibitors of the HIF pathway and synthetic lethal interactions. Expert Opin Ther Targets.

[119] Seo B.R., Min K.J., Cho I.J., Kim S.C., Kwon T.K. (2014). Curcumin significantly enhances dual PI3K/Akt and mTOR inhibitor NVP-BEZ235-induced apoptosis in human renal carcinoma Caki cells through down-regulation of p53-dependent Bcl-2 expression and inhibition of Mcl-1 protein stability. PLoS One.

[120] Hers I., Vincent E.E., Tavaré J.M. (2011). Akt signalling in health and disease. Cell Signal.

[121] Wouters B.G., Koritzinsky M. (2008). Hypoxia signalling through mTOR and the unfolded protein response in cancer. Nat Rev Cancer.

[122] Lee S.-H., Jee J.-G., Bae J.-S., Liu K.-H., Lee Y.M. (2015). A group of novel HIF-1α inhibitors, glyceollins, blocks HIF-1α synthesis and decreases its stability via inhibition of the PI3K/AKT/mTOR pathway and Hsp90 binding. J Cell Physiol.

[123] Creighton-Gutteridge M., Cardellina J.H., Stephen A.G. (2007). Cell type–specific, topoisomerase II–dependent inhibition of hypoxia-inducible factor-1α protein accumulation by NSC 644221. Clin Cancer Res.

[124] Naldini A., Filippi I., Cini E., Rodriquez M., Carraro F., Taddei M. (2012). Downregulation of hypoxia-related responses by novel antitumor histone deacetylase inhibitors in MDAMB231 breast cancer cells. Anti Cancer Agents Med Chem.

[125] Zhou Q., Gustafson D., Nallapareddy S. (2011). A phase I dose-escalation, safety and pharmacokinetic study of the 2-methoxyestradiol analog ENMD-1198 administered orally to patients with advanced cancer. Invest N Drugs.

[126] Nepal M., Gong Y.-D., Park Y.R., Soh Y. (2011). An activator of PHD2, KRH102140, decreases angiogenesis via inhibition of HIF-1α. Cell Biochem Funct.

[127] Hirota K., Semenza G.L. (2006). Regulation of angiogenesis by hypoxia-inducible factor 1. Crit Rev Oncol Hematol.

[128] Ferrara N. (2005). The role of VEGF in the regulation of physiological and pathological angiogenesis. Birkhäuser Basel.

[129] Cohen M.H., Shen Y.L., Keegan P., Pazdur R. (2009). FDA drug approval summary: Bevacizumab (Avastin) as treatment of recurrent glioblastoma multiforme. Oncologist.

[130] Busk M., Walenta S., Mueller-Klieser W. (2011). Inhibition of tumor lactate oxidation: Consequences for the tumor microenvironment. Radiother Oncol.

[131] Koh M.Y., Spivak-Kroizman T., Venturini S. (2008). Molecular mechanisms for the activity of PX-478, an antitumor inhibitor of the hypoxia-inducible factor-1α. Mol Cancer Therapeut.

[132] Kane R.C., Bross P.F., Farrell A.T., Pazdur R. (2003). Velcade: U.S. FDA approval for the treatment of multiple myeloma progressing on prior therapy. Oncologist.

[133] Campbell S.L., Wellen K.E. (2018). Metabolic signaling to the nucleus in cancer. Mol Cell.

[134] Meng L., Cheng Y., Tong X. (2018). Tumor oxygenation and hypoxia inducible factor-1 functional inhibition via a reactive oxygen species responsive nanoplatform for enhancing radiation therapy and abscopal effects. ACS Nano.

[135] Okuno T., Kawai K., Hata K. (2018). SN-38 acts as a radiosensitizer for colorectal cancer by inhibiting the radiation-induced up-regulation of HIF-1α. Anticancer Res.

[136] Xie T., Wang J.R., Dai C.G., Fu X.A., Dong J., Huang Q. (2020). Vitexin, an inhibitor of hypoxia-inducible factor-1α, enhances the radiotherapy sensitization of hyperbaric oxygen on glioma. Clin Transl Oncol.

[137] Lee J.H., Shim J.W., Choi Y.J., Heo K., Yang K. (2013). The combination of sorafenib and radiation preferentially inhibits breast cancer stem cells by suppressing HIF-1α expression. Oncol Rep.

[138] Pore N., Gupta A.K., Cerniglia G.J. (2006). Nelfinavir down-regulates hypoxia-inducible factor 1α and VEGF expression and increases tumor oxygenation: Implications for radiotherapy. Cancer Res.

[139] Wu Y., Zheng Y., Shen Z., Ge W., Xie Y., Li C. (2014). Endostar combined with radiotherapy increases radiation sensitivity by decreasing the expression of TGF-β1, HIF-1α and bFGF. Exp Ther Med.

[140] Jafari S., Dabiri S., Mehdizadeh Aghdam E. (2023). Synergistic effect of chrysin and radiotherapy against triple-negative breast cancer (TNBC) cell lines. Clin Transl Oncol.

[141] Ashton T.M., Fokas E., Kunz-Schughart L.A. (2016). The anti-malarial atovaquone increases radiosensitivity by alleviating tumour hypoxia. Nat Commun.

[142] Ghimessy Á.K., Gellert Á., Schlegl E. (2019). KRAS mutations predict response and outcome in advanced lung adenocarcinoma patients receiving first-line bevacizumab and platinum-based chemotherapy. Cancers.

[143] Sun D-c, Shi Y., Wang Y-r (2017). KRAS mutation and primary tumor location do not affect efficacy of bevacizumab-containing chemotherapy in stagae IV colorectal cancer patients. Sci Rep.

[144] Torok S., Rezeli M., Kelemen O. (2017). Limited tumor tissue drug penetration contributes to primary resistance against angiogenesis inhibitors. Research Paper. Theranostics.

[145] Mbofung R.M., McKenzie J.A., Malu S. (2017). HSP90 inhibition enhances cancer immunotherapy by upregulating interferon response genes. Nat Commun.

[146] Wanfeng G., Xiaoyun Z., Wendong Y. (2019). Prim-O-glucosylcimifugin enhances the antitumour effect of PD-1 inhibition by targeting myeloid-derived suppressor cells. J Immunother Cancer.

[147] Kao T.-W., Bai G.-H., Wang T.-L. (2023). Novel cancer treatment paradigm targeting hypoxia-induced factor in conjunction with current therapies to overcome resistance. J Exp Clin Cancer Res.

[148] Ikezawa Y., Sakakibara-Konishi J., Mizugaki H., Oizumi S., Nishimura M. (2017). Inhibition of Notch and HIF enhances the antitumor effect of radiation in Notch expressing lung cancer. Int J Clin Oncol.

[149] Kazimova T., Tschanz F., Sharma A. (2021). Paracrine placental growth factor signaling in response to ionizing radiation is p53-dependent and contributes to radioresistance. Mol Cancer Res.

[150] Schuler P.J., Harasymczuk M., Schilling B. (2013). Effects of adjuvant chemoradiotherapy on the frequency and function of regulatory T cells in patients with head and neck cancer. Clin Cancer Res.

[151] Sharabi A., Nirschl C., Ceccato T. (2014). Role of radiation therapy in inducing antigen specific antitumor immune responses when combined with anti-PD1 checkpoint blockade: Mechanism and clinical implications. Int J Radiat Oncol Biol Phys.

[152] Ding X-c, Wang L-l, Zhang X-d (2021). The relationship between expression of PD-L1 and HIF-1α in glioma cells under hypoxia. J Hematol Oncol.

[153] Wang Y., Liu Z.-G., Yuan H. (2019). The reciprocity between radiotherapy and cancer immunotherapy. Clin Cancer Res.

[154] Cobleigh M.A., Langmuir V.K., Sledge G.W. (2003). A phase I/II dose-escalation trial of bevacizumab in previously treated metastatic breast cancer. Semin Oncol.

[155] Semenza Gregg L. (2012). Hypoxia-inducible factors in physiology and medicine. Cell.

[156] Gong P.-J., Shao Y.-C., Huang S.-R. (2020). Hypoxia-associated prognostic markers and competing endogenous RNA co-expression networks in breast cancer. Front Oncol.

[157] Folkman J. (2003). Angiogenesis and apoptosis. Semin Cancer Biol.

[158] Harris A.L. (2002). Hypoxia—a key regulatory factor in tumour growth. Nat Rev Cancer.

[159] Azad S.M.A.K., Meem J.N., Shaikat A.H. (2024). Targeting STAT3-mediated autophagy with small molecules in cancer treatment – a comprehensive review. Clin Tradit Med Pharmacol.

[160] Gouel P., Decazes P., Vera P., Gardin I., Thureau S., Bohn P. (2023). Advances in PET and MRI imaging of tumor hypoxia. Front Med.

[161] Acharya S., Misra R. (2022). Hypoxia responsive phytonanotheranostics: A novel paradigm towards fighting cancer. Nanomedicine (N Y).

[162] O'Connor J.P., Robinson S.P., Waterton J.C. (2019). Imaging tumour hypoxia with oxygen-enhanced MRI and BOLD MRI. Br J Radiol.

[163] Chen J., Lanza G.M., Wickline S.A. (2010). Quantitative magnetic resonance fluorine imaging: today and tomorrow. Wiley Interdiscip Rev Nanomed Nanobiotechnol.

[164] Godet I., Doctorman S., Wu F., Gilkes D.M. (2022). Detection of hypoxia in cancer models: Significance, challenges, and advances. Cells.

[165] Zarrer J., Taipaleenmäki H. (2024). The osteoblast in regulation of tumor cell dormancy and bone metastasis. J Bone Oncol.

[166] Kim S.J., Rabbani Z.N., Dewhirst M.W. (2005). Expression of HIF-1α, CA IX, VEGF, and MMP-9 in surgically resected non-small cell lung cancer. Lung Cancer.

[167] Knowles H.J. (2015). Hypoxic regulation of osteoclast differentiation and bone resorption activity. Hypoxia.

[168] Cao D., Hou M., Guan Y-s, Jiang M., Yang Y., Gou H-f (2009). Expression of HIF-1alpha and VEGF in colorectal cancer: Association with clinical outcomes and prognostic implications. BMC Cancer.

[169] Dai Y., Bae K., Siemann D.W. (2011). Impact of hypoxia on the metastatic potential of human prostate cancer cells. Int J Radiat Oncol Biol Phys.

[170] Krock B.L., Skuli N., Simon M.C. (2011). Hypoxia-induced angiogenesis: Good and evil. Genes Cancer.

[171] Nardinocchi L., Puca R., Sacchi A., D'Orazi G. (2009). Inhibition of HIF-1alpha activity by homeodomain-interacting protein kinase-2 correlates with sensitization of chemoresistant cells to undergo apoptosis. Mol Cancer Res.

[172] Chen L., Feng P., Li S. (2009). Effect of hypoxia-inducible factor-1α silencing on the sensitivity of human brain glioma cells to doxorubicin and etoposide. Neurochem Res.

[173] Sasabe E., Zhou X., Li D., Oku N., Yamamoto T., Osaki T. (2007). The involvement of hypoxia-inducible factor-1α in the susceptibility to γ-rays and chemotherapeutic drugs of oral squamous cell carcinoma cells. Int J Cancer.

[174] Liu L., Ning X., Sun L. (2008). Hypoxia-inducible factor-1α contributes to hypoxia-induced chemoresistance in gastric cancer. Cancer Sci.

[175] Li J., Shi M., Cao Y. (2006). Knockdown of hypoxia-inducible factor-1α in breast carcinoma MCF-7 cells results in reduced tumor growth and increased sensitivity to methotrexate. Biochem Biophys Res Commun.

[176] Wirthner R., Wrann S., Balamurugan K., Wenger R.H., Stiehl D.P. (2008). Impaired DNA double-strand break repair contributes to chemoresistance in HIF-1α-deficient mouse embryonic fibroblasts. Carcinogenesis.

[177] Unruh A., Ressel A., Mohamed H.G. (2003). The hypoxia-inducible factor-1α is a negative factor for tumor therapy. Oncogene.

[178] Sullivan R., Graham C.H. (2009). Hypoxia prevents etoposide-induced DNA damage in cancer cells through a mechanism involving hypoxia-inducible factor 1. Mol Cancer Therapeut.

[179] Courtney K.D., Infante J.R., Lam E.T. (2018). Phase I dose-escalation trial of PT2385, a first-in-class hypoxia-inducible factor-2α antagonist in patients with previously treated advanced clear cell renal cell carcinoma. J Clin Oncol.

[180] Nickols N.G., Jacobs C.S., Farkas M.E., Dervan P.B. (2007). Modulating hypoxia-inducible transcription by disrupting the HIF-1–DNA interface. ACS Chem Biol.

[181] Jensen R.L., Ragel B.T., Whang K., Gillespie D. (2006). Inhibition of hypoxia inducible factor-1α (HIF-1α) decreases vascular endothelial growth factor (VEGF) secretion and tumor growth in malignant gliomas. J Neuro Oncol.

[182] Karni-Schmidt O., Lokshin M., Prives C. (2016). The roles of MDM2 and MDMX in cancer. Annu Rev Pathol.

[183] Talekar M., Tran T.-H., Amiji M. (2015). Translational nano-medicines: Targeted therapeutic delivery for cancer and inflammatory diseases. AAPS J.

[184] Shimizu T., Okamoto I., Tamura K. (2010). Phase I clinical and pharmacokinetic study of the glucose-conjugated cytotoxic agent D-19575 (glufosfamide) in patients with solid tumors. Cancer Chemother Pharmacol.

[185] Janowska S., Paneth A., Wujec M. (2020). Cytotoxic properties of 1,3,4-thiadiazole derivatives—A review. Molecules.

[186] Razak M.A.I.A., Hamid H.A., Othman R.N.I.R., Moktar S.A.M., Miskon A. (2021). Improved drug delivery system for cancer treatment by d-glucose conjugation with eugenol from natural product. Curr Drug Deliv.

[187] Miranda-Gonçalves V., Gonçalves C., Granja S. (2021). MCT1 is a new prognostic biomarker and its therapeutic inhibition boosts response to temozolomide in human glioblastoma. Cancers (Basel).

[188] Ocaña M.C., Martínez-Poveda B., Marí-Beffa M., Quesada A.R., Medina M.Á. (2020). Fasentin diminishes endothelial cell proliferation, differentiation and invasion in a glucose metabolism-independent manner. Sci Rep.

[189] Gubens M.A., Davies M. (2019). NCCN guidelines updates: New immunotherapy strategies for improving outcomes in non–small cell lung cancer. J Natl Compr Cancer Netw.

[190] Mielczarek-Lewandowska A., Hartman M.L., Czyz M. (2020). Inhibitors of HSP90 in melanoma. Apoptosis.

[191] Luo Y., Zeng R., Guo Q., Xu J., Sun X., Wang L. (2019). Identifying a novel anticancer agent with microtubule-stabilizing effects through computational cell-based bioactivity prediction models and bioassays. Org Biomol Chem.

[192] Leung E., Cairns R.A., Chaudary N. (2017). Metabolic targeting of HIF-dependent glycolysis reduces lactate, increases oxygen consumption and enhances response to high-dose single-fraction radiotherapy in hypoxic solid tumors. BMC Cancer.

[193] Xiang G.-L., Zhu X.-H., Lin C.-Z. (2017). 125I seed irradiation induces apoptosis and inhibits angiogenesis by decreasing HIF-1α and VEGF expression in lung carcinoma xenografts. Oncol Rep.

[194] Weichselbaum R.R., Liang H., Deng L., Fu Y.-X. (2017). Radiotherapy and immunotherapy: A beneficial liaison?. Nat Rev Clin Oncol.

[195] Tian L., Goldstein A., Wang H. (2017). Mutual regulation of tumour vessel normalization and immunostimulatory reprogramming. Nature.

